# Wearable Sensors and Smart Devices to Monitor Rehabilitation Parameters and Sports Performance: An Overview

**DOI:** 10.3390/s23041856

**Published:** 2023-02-07

**Authors:** Roberto De Fazio, Vincenzo Mariano Mastronardi, Massimo De Vittorio, Paolo Visconti

**Affiliations:** 1 Department of Innovation Engineering, University of Salento, 73100 Lecce, Italy; 2 Facultad de Ingeniería, Universidad Panamericana, Aguascalientes 20290, Mexico; 3 Center for Biomolecular Nanotechnologies, Italian Technology Institute IIT, 73010 Arnesano, Italy

**Keywords:** advanced diagnostics, bio-vital markers, e-healthcare, injury-prevention, mini-invasive monitoring, rehabilitation parameters, sports performance tracking, wearable sensors

## Abstract

A quantitative evaluation of kinetic parameters, the joint’s range of motion, heart rate, and breathing rate, can be employed in sports performance tracking and rehabilitation monitoring following injuries or surgical operations. However, many of the current detection systems are expensive and designed for clinical use, requiring the presence of a physician and medical staff to assist users in the device’s positioning and measurements. The goal of wearable sensors is to overcome the limitations of current devices, enabling the acquisition of a user’s vital signs directly from the body in an accurate and non–invasive way. In sports activities, wearable sensors allow athletes to monitor performance and body movements objectively, going beyond the coach’s subjective evaluation limits. The main goal of this review paper is to provide a comprehensive overview of wearable technologies and sensing systems to detect and monitor the physiological parameters of patients during post–operative rehabilitation and athletes’ training, and to present evidence that supports the efficacy of this technology for healthcare applications. First, a classification of the human physiological parameters acquired from the human body by sensors attached to sensitive skin locations or worn as a part of garments is introduced, carrying important feedback on the user’s health status. Then, a detailed description of the electromechanical transduction mechanisms allows a comparison of the technologies used in wearable applications to monitor sports and rehabilitation activities. This paves the way for an analysis of wearable technologies, providing a comprehensive comparison of the current state of the art of available sensors and systems. Comparative and statistical analyses are provided to point out useful insights for defining the best technologies and solutions for monitoring body movements. Lastly, the presented review is compared with similar ones reported in the literature to highlight its strengths and novelties.

## 1. Introduction

Monitoring human physiological functions and performance during real-time activities is becoming increasingly popular in sporting and healthcare environments [1,2,3,4]. Recent studies demonstrated that introducing enhanced recovery after surgery programs can significantly improve post-surgical recovery quality, thereby helping to decrease the time for rehabilitation outside clinical centers [5,6]. Traditionally, doctors or physicians assist the patients in completing the rehabilitation training using hands or basic and standard equipment. The assessment of a patient’s physical state, the degree of rehabilitative training, and the effectiveness of the rehabilitation results are thus highly dependent on the physician’s expertise and level. Moreover, more than 70% of patients usually fail to comply with post-surgical programs when moving home. The main limitations to a successful rehabilitation are the lack of time and feedback from specialists, the difficulties in remembering how to execute the exercises, and the inability to feel the benefits of the therapies and the related improvements. Therefore, the measurement of a human being’s kinetic and other vital and physiological parameters is essential to monitor and collect a series of data (e.g., movements, bending, joint rotation, and so on) useful to provide important tracking feedback to the user. This allows for the adoption of suitable protocols that ensure the patients carry out their physiotherapy programs even at home. In rehabilitation after trauma or injuries, these data allow for defining the correct therapies and evaluating their effectiveness and patient progress. Moreover, in sports training, they allow for optimizing the exercises and assessing an athlete’s progress.

The rapid development of the Internet of things [7,8,9], due to the technological advancement of sensor and communication technology, together with increased availability, lower cost, and advancements in personal computing devices, has paved the way for new opportunities and challenges for the research and development of wearable healthcare devices [7,8,10,11,12,13,14]. Wearable sensors enable users to identify and measure different clinically relevant parameters, such as functional motions, biomechanical and bio–vital parameters, and athletes’ workloads, to enhance performance while reducing the risk of injury. Wearable monitoring devices can give a continuous and real-time flow of physiological data, creating precise treatment plans and player-specific training regimens to help minimize or prevent injuries.

This review aims to provide an overview of the most recent wearable sensors and systems in the literature used to detect and monitor physiological parameters in athlete training tracking and post-operative rehabilitation of patients. At first, the most significant parameters for monitoring post-operative rehabilitation and sports performances are introduced. Additionally, a first classification of the discussed and analyzed parameters is provided, distinguishing between medical rehabilitation and sports fields. After this, an analysis of the available technologies for detecting and acquiring the physiological parameters directly from the human body is provided. In detail, wearable sensors will be classified according to the parameters they allow to identify, such as body movements or physiological parameters. Inertial, capacitive, piezoresistive, piezoelectric, and flexoelectric transduction mechanisms will be detailed. Afterward, some figures of merit (FoM) helpful for classifying the analyzed technologies in terms of performance are shortly described. FoM are used in this paper to identify the characteristic parameters of the discussed technologies and compare and classify the working mechanisms used to implement the analyzed transduction mechanisms. These quantities allow quantifying their efficiency and performance. An overview of wearable technologies for monitoring body motions is presented, providing a detailed depiction of the available sensors and systems. Additionally, they are classified in two main categories: (i) wearable sensors for post-operative rehabilitation monitoring and (ii) wearable sensors for athletes’ performance monitoring. The described technologies are then compared to achieve a detailed classification according to the sensor’s maturity, sensitivity, the range of detection, the area of the body where they are typically placed, the measured parameters allowed by that specific technology, and the corresponding response time. In addition, several publicly available datasets related to sports and rehabilitation monitoring are discussed. Finally, a comparison of the presented review work with similar ones reported in the scientific literature is presented to highlight its novelties and strengths. The main novelties and contributions of the presented review are:

- A detailed classification of human physiological parameters useful for extracting information about a patient’s health status during post-operative rehabilitation and sports performances.
- An in-depth analysis of the transduction mechanisms for acquiring parameters related to body motions; also, innovative wearable technologies and sensors to monitor human activities are discussed, ranked by high comfort and flexibility.
- A comprehensive overview of available wearable technologies for monitoring motions of body parts and physiological parameters to enhance rehabilitation therapies and customize athletes’ training.
- A comparison of the presented review with similar ones reported in the literature; its strengths lie in its completeness and level of detail, dealing with both sensing systems for monitoring rehabilitation and sports performances. In addition, it does not limit the discussion to specific sensor categories, applications, or monitored body areas, as detailed in the comparison reported in Section 6. The joint discussion of the two applications represents one of the novelties of the presented work, rarely treated in other review articles with the presented level of detail. In addition, the proposed work reports comparative analyses related to the discussed scientific studies from the performance point of view, providing useful insights for determining the best sensing strategies for developing future wearable systems for monitoring the human body.

The remainder of the review is arranged as follows: Section 2 presents a detailed classification of the primary parameters acquired on the body surface to extract information related to post-operative rehabilitation or sports training. Afterward, Section 3 analyzes the main sensing mechanisms for detecting body motions related to physiological activities; later, the figures of merit for comparing and classifying sensing mechanisms implemented by wearable devices are described. Section 5 reports a survey of available wearable technologies for monitoring body motions which find application in post-operative rehabilitation and athlete performance tracking. Furthermore, an overview of datasets related to sports and rehabilitation monitoring is introduced. Lastly, Section 6 presents a comparative analysis of the presented review work with similar ones reported in the literature, highlighting its strengths and novelties.

### Selection and Exclusion Criteria for the Presented Review Paper

**Figure 1:**
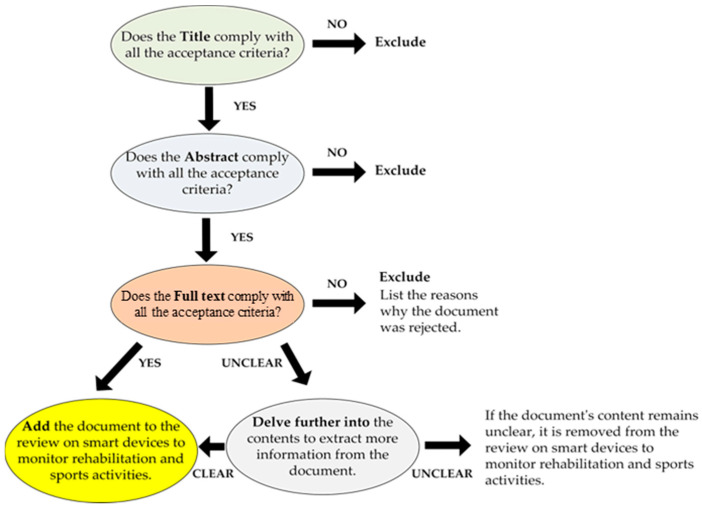
Workflow describing the selection process involved in the presented review paper.

Before starting the discussion of the presented review is necessary to define the criteria used to select and exclude the most appropriate scientific works and review papers. The latter has been defined specifically, taking into account many elements of the analyzed documents, such as applicability to the treated themes, relevance, publication year, and redundancy concerning other chosen articles. The goal was to provide the reader with as broad a view as possible of wearable systems and technologies for monitoring body motions and biophysical parameters which assist medical staff in a patient’s rehabilitation process and tracking an athlete’s performances. The selection process was conducted following the methodology shown in Figure 1; in particular, analysis of the documents was performed according to a three-step procedure. In detail, the procedure began with the title’s evaluation, followed by the analysis of the abstract, then coming to the careful reading of the full paper. Each step involved a binary evaluation, leading to acceptance or rejection of the considered document according to the criteria above (Figure 1). At the end of the analysis, if the document was unclear, analysis of its contents was further deepened by researching information from external sources. If, after this study, the contents remained unclear, the document was discarded from the review, noting the reasons for its exclusion.

**Figure 2:**
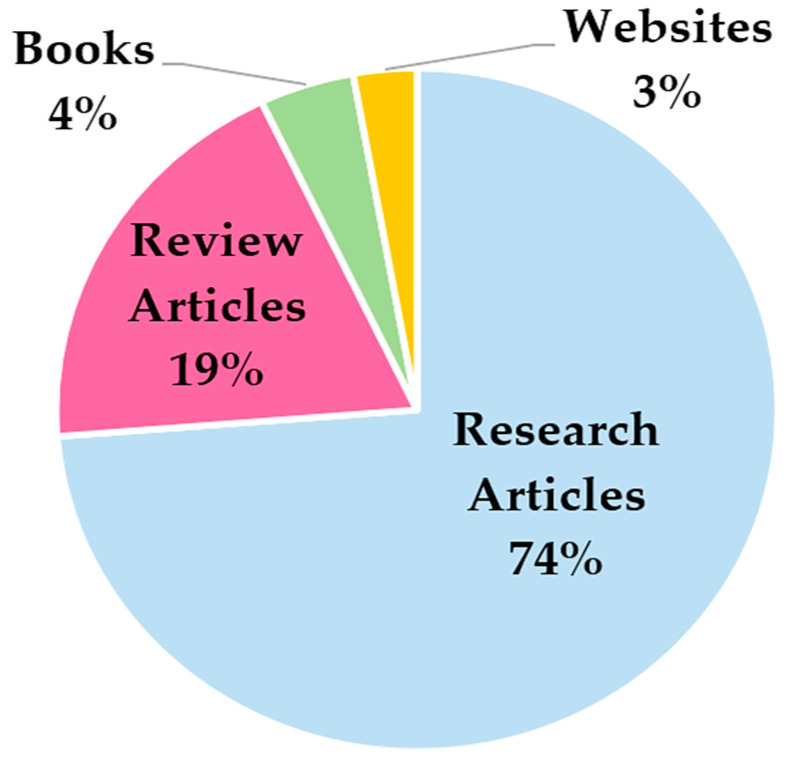
Distribution of the documents selected for structuring the presented review paper on smart devices for monitoring rehabilitation and tracking sports activities.

Ultimately, 141 documents were analyzed to realize the overview of smart devices for monitoring rehabilitation and tracking sports activities; Figure 2 depicts the distribution of the selected documents grouped according to their typology (research articles, review articles, and books).

**Figure 3:**
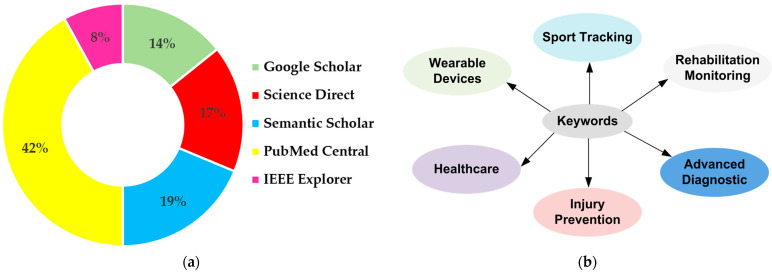
Distribution of the databases (**a**) and main keywords (**b**) used for searching the documents included in this review.

Furthermore, Figure 3 reports the distribution of the databases and the main keywords used to research the documents included in the presented review.

## 2. Classification of Human Physiological Parameters

The measurement of vital human parameters is essential to provide important tracking insights and feedback on the health status of the final user. For this reason, the acquisition of information directly from the human body by sensors attached to sensitive skin locations or worn as a part of garments allow obtaining clinically relevant parameters. They ensure a continuous flow of data, going beyond the limits of traditional rehabilitation and training methods [15].

Some joints, such as the elbow, are limited to bending in one direction; others, such as the shoulders and hip, can perform more complex movements in three dimensions. In this respect, one of the relevant key parameters that should be monitored during rehabilitation after injury to the bones, muscles, or nerves is the range of motion (*ROM*) [16,17,18]. Being calculated as the joint’s rotation angle in respect to a reference plane, as in the case for elbow flexion/extension, or a reference axis, as in forearm pronation/supination movements, it is defined as the freedom of joint movements in a space (Table 1). It is obtained by measuring the angle between the standard joint position and the final one the patient can reach without experiencing pain; thus, it is a suitable parameter to evaluate improvements over time [19,20,21,22,23].

**Figure 4:**
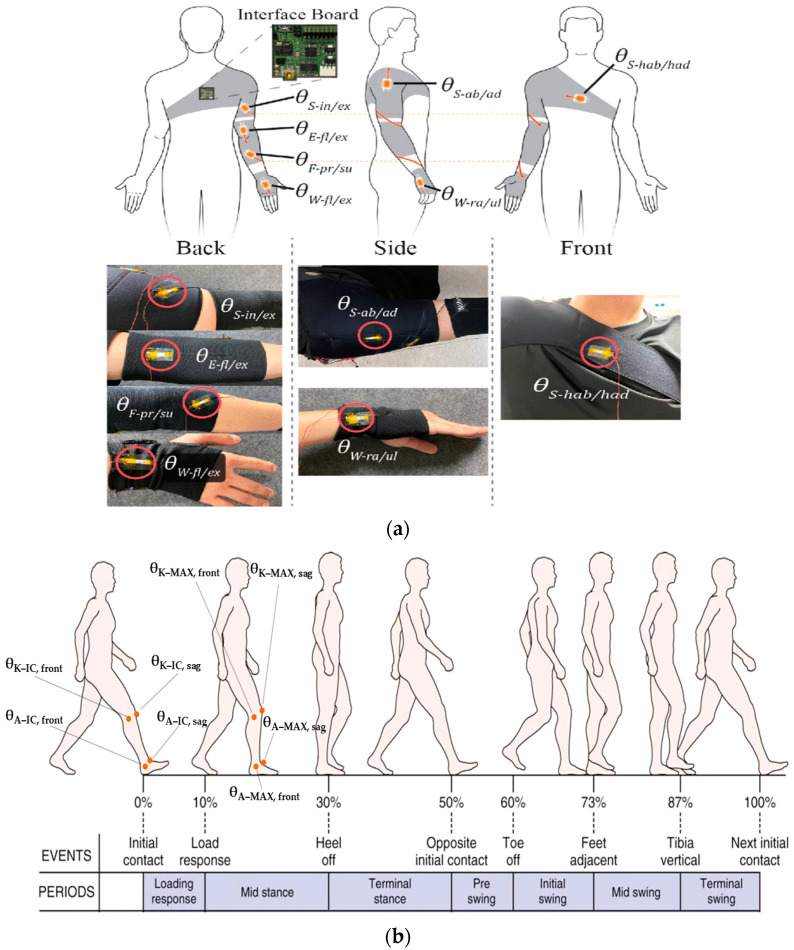
Schematic illustration of a subject wearing tendon-inspired motion detection devices and pictures of the integrated device into wearable belts (**a**) [24]. The main angles measured on the lower limbs for rehabilitation monitoring and sports tracking are highlighted in (**b**).

In this context, Kim et al. in [24] analyzed patients’ upper joint movement ranges by using wearable devices. As a monitoring benchmark, the authors calculated the angles of different upper joints. The wrist flexion/extension, wrist radial/ulnar deviation, shoulder external/internal rotation, shoulder adduction/abduction, forearm supination/pronation, and elbow flexion/extension were then studied by tracking the detected angles, enabling to emulate each patient’s movements using a real-time animation of an avatar to improve the monitoring experience (Figure 4a). An additional parameter that allows evaluating a patient’s improvements during rehabilitation is the comparison between the mobility range of the limb affected by the trauma and the mobility of a healthy one—e.g., how much the injured limb is used during the day—establishing the movement frequency. Together with the parameters above, the patient’s balance and gait [25,26,27], cardiac and respiratory parameters (such as HR—heart rate, blood pressure, HRV—heart rate variability, RR—respiration rate, etc.) [28,29,30,31], neck muscle vibrations (frequency, pattern, intensity) [32], chewing (duration and frequency) [21], and swallowing frequency [33,34] are recorded. The latest, especially in patients affected by diseases such as dysphasia and dysphagia, are studied and analyzed to monitor the patient’s health after surgery and understand how some pathologies affect their normal state.

As reported in Table 1, other fundamental angles can be extracted for the lower limb. The knee and ankle angles are essential for preventing injuries; in particular, the initial contact angle (IC), maximum angle (MAX) at the midstance, as well as the latency between the IC and MAX can be extracted both in the frontal and sagittal planes (Figure 4b) [35]. These parameters are subject to significant variations during running over long distances, and are also affected by the typology of worn footwear [35]. Furthermore, from the analysis of kinetic data, the elastic characteristics of ligament structures in the knee extensors and plantar flexors can be calculated [36].

As with the post-operative rehabilitation process, it is also possible to identify some key physiological parameters for monitoring athletes’ sports performance. Some of these parameters can overlap with those mentioned above: for example, ROM and joint motion angles can provide meaningful feedback on the definition of movement strategies for athletes or the correct execution of motion in a specific discipline [37,38,39,40,41,42]. Intensity and trajectory of body movements, in addition to heart and breathing parameters (e.g., HR, SpO_2_, RR, etc.) during long physical activity periods, can carry information about an athlete’s fatigue and fitness decrease [10], which provide essential feedback on the athlete’s health status. Correct execution of running or jumping [43,44,45], hit pressure [46] in combat sports, response time, and acceleration to execute a movement [47] are additional parameters of interest that are usually monitored during sports activity tracking [48,49,50,51,52]. S. Saponara designed and proposed a wearable device capable of monitoring some of an athlete’s key movement parameters in combat sports [47]. In this work, the author analyzed the pressure of kicks and punches on a target body to quantify a movement’s effectiveness from a traumatological model correlated with the subjective indication of a coach. The acceleration of movements was also analyzed and compared with the ideal values for that specific technique, allowing the athlete to avoid trajectories that deviate from the standard. Additionally, the user’s response time was studied to examine the time to pull a kick or a punch—roughly a tenth of second—depending on the combat technique and athlete’s physical characteristics.

A classification of the parameters (or group of parameters) discussed above is summarized in Table 2, where medical rehabilitation and sports fields were distinguished; this classification derives from the analyses in the scientific literature reported in this review work. However, some parameters are involved both in rehabilitation monitoring and sports performance tracking, such as heart and breathing parameters. This classification aims to give the reader a comprehensive overview of the main parameters monitored during medical rehabilitation and sports activities.

**Figure 5:**
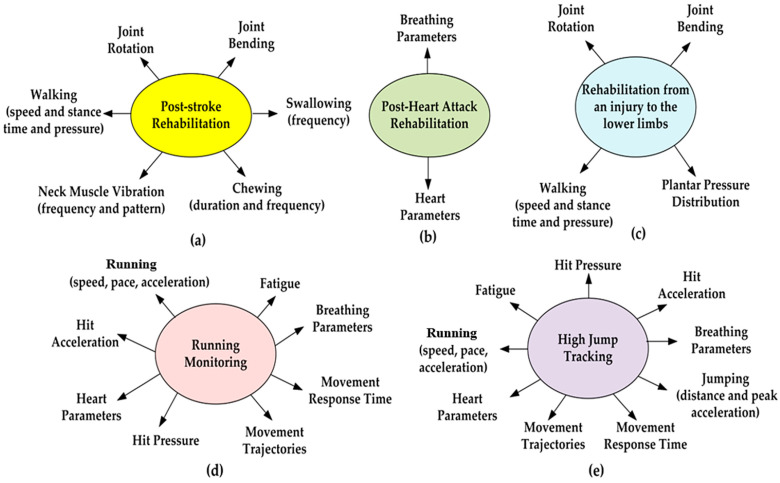
Plots representing the parameters and gestures monitored for the different typologies of clinical rehabilitation: post-stroke (**a**), post-heart attack (**b**) and rehabilitation from an injury to the lower limbs (**c**). Parameters and gestures monitored in sports activity: running monitoring (**d**) and high jump tracking (**e**).

In conclusion, considering the parameters and physiological processes reported in Table 2, the set measured depends on the typology of clinical rehabilitation or sports activity to be monitored. For instance, Figure 5 depicts some examples with related monitored parameters and activities.

## 3. Transduction Mechanisms for the Acquisition of Human Parameters from the Body

In the last decade, the advancement of various sensing technologies and the development of highly flexible and conformable wearable devices and sensors have paved the way for innovative solutions that monitor human activities by exploiting minimally invasive and comfortable devices.

In broad terms, wearable sensors can be classified by considering the parameters they should identify: body movements (position, gait, acceleration, etc.) or physiological parameters (heart rate, heart rate variability, voice, etc.). However, a more detailed classification is based on the working transduction mechanism. In this respect, inertial, optical, and angular sensors are the main instruments for monitoring the human body’s gait.

Optical fiber sensors (OFS) are based on optical technology and do not suffer from electromagnetic interference. OFS are composed of a light source that transmits a light beam to a photodetector through an optical fiber. They can be used to assess a joint’s bending angles by measuring the angle and the attenuation of the reflected light beam when detected by the photodetector [65,66,67,68]. Joint motion angles are also measured using angular sensors or goniometers based on strain gauges or resistive potentiometers. However, some of the main problems of these sensors are their lower accuracy and rigidity, which do not allow them to be positioned comfortably on the joints. Markerless and marker-based motion capture technologies are also available for monitoring human physiological parameters. They are used to quantify the kinematics of a motion, with the ability to enhance clinical evaluations of function and performance [25,69,70,71,72,73,74].

**Figure 6:**
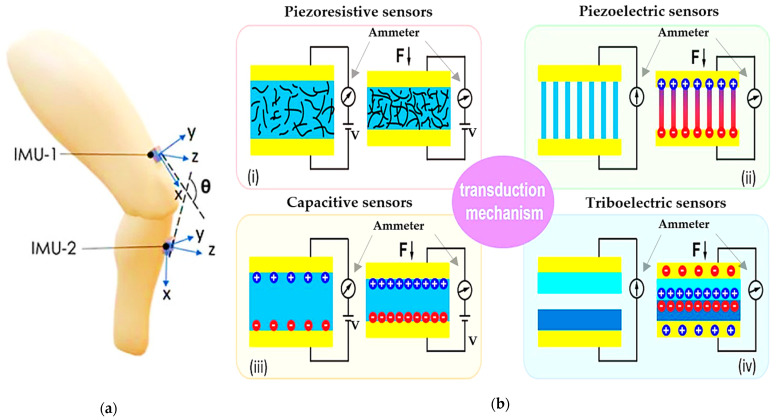
Schematic illustration of (**a**) inertial sensors (“reproduced with permission from S. Z. Homayounfar et al., SLAS Technology; published by Elsevier, 2020” [79]); (**b**) piezoresistive (**i**), piezoelectric (**ii**), capacitive (**iii**), and triboelectric (**iv**) sensors [80]).

An alternative to the previously mentioned sensors, inertial sensors, are widely used by clinicians to perform kinematic measurements to monitor both healthy and pathological movements, quantify the degree of impairment and the severity of the damage, plan rehabilitation strategies, and evaluate the impact of various therapies. An inertial sensor consists of a small and rigid central body, the inertial measurement unit (IMU), which usually integrates MEMS devices such as accelerometers, gyroscopes, and magnetometers, enabling the perception of movement in multiple dimensions using a single sensor. They are usually placed above and below the joints (neck, fingers, elbow, shoulder, hip, knee, ankle, etc.), and they allow detection of bending angle, linear 3D acceleration, three-dimensional orientation, and angular velocity to track joint movements, walking speed, etc. (Figure 6a) [75,76,77,78]. Moreover, an additional advantage of IMU is the capacity to measure a patient’s or an athlete’s energy consumption—critical for determining, for example, the intensity of the training—by multiple integrations of vertical acceleration over time. Despite their high accuracy, low-cost design, and portability, IMU sensors are sensitive to electromagnetic interference and noise, especially indoors; they are affected by a drift effect and high rigidity, limiting their daily application. For this reason, their utility is often restricted to clinical applications under expert supervision.

Electromechanical sensors based on different transduction methods, such as capacitive, triboelectric, piezoresistive, and piezoelectric effects, are more appropriate for wearable applications. By virtue of the material used for their fabrication, these sensors are usually lighter, comfortable, robust, and suitable to detect physiological parameters directly from the human body since they can be attached to or integrated into clothes, wraps, yarns, etc. [81]. These devices can be fruitful in several healthcare applications, covering continuous health monitoring, daily and athletic activity tracking, and acting as a multifunctional electronic skin. However, the employment of nanomaterial-enabled wearable sensors on a large scale faces several challenges and technical issues, such as improving their performance (biocompatibility, performance stability, multi-modal sensing capability), efficient integration, and advances of other compliant/stretchable components.

Piezoresistive sensors (Figure 6b-i) are the most widely used technologies to transform deformation stimuli into electrical signals. The working principle of these sensors relies on the relation

(1) $$R=\frac{\rho l}{A}$$

where l is the surface’s length, A the cross-section’s area, and ρ the material’s electrical resistivity. By choosing suitable materials, and varying one or more of these parameters by deforming the structure of the sensor, the resistance value changes accordingly. By calibrating the sensor and correlating the resistance variation to the applied compression or traction force, it is possible to evaluate the intensity and magnitude of the external stimuli. Piezoresistive sensors can be classified into strain and pressure sensors depending on the mechanical force applied. The choice of suitable materials, the production process, and the general operation of piezoresistive strain sensors have been extensively studied by researchers.

Moreover, structured 3D patterns, such as micro-pillars, half-spheres, or pyramid shapes, can affect the performance of piezoresistive sensors, as well as the combination of conductive materials such as carbon nanotubes (CNTs), cellulose nanocrystals (CNCs), silver nanowires (AgNWs), graphene, and other polymers, with a rubber body to provide elastic properties. If subjected to a tensile force, the resulting compound varies the conductive paths, redistributing the percentage of the conductivity inside the material and, consequently, varying the resistance value. An interesting fabrication method involves synthesizing the piezoresistive material as a fabric in clothes and optimizing its functionality, for example using elastic fibers painted with conductive substances or wool fibers covered with graphene nano-sheets (GNPs) or conductive carbon (CB) [27,30,32,56,82]. Yao et al. in [83] presented a novel methodology for integrating multifunctional e-textiles without sacrificing stretchability, wearability, or washability. The material was obtained by depositing from a AgNW solution onto a glass plane and evaporating the residual solvent. After, a TPU layer was spin-coated over the AgNW network, letting the solvent evaporate and solidify. Then, the layer was transferred onto a stretchable fabric by heat-pressing at 140 °C. The test results indicated that the resulting AgNW–TPU layer is characterized by low sheet resistance (0.2 Ω/□), a low Young’s modulus, excellent stretchability (50%), and good washability (10% resistance increase after 100 washing cycles).

Capacitive sensors exploit the physical properties of a capacitor made of proper elastic materials, to vary the capacitance value when pressure or traction is applied (Figure 6b-ii). They consist of two metal electrodes separated by a dielectric elastic material, such that the capacitance is

(2) $$C=\frac{\varepsilon_{0}\varepsilon_{r}A}{d}$$

where A is the surface area of the two metal plates, d is the thickness of the dielectric material between them, ε_0_ is the electrical permittivity of the vacuum, and ε_r_ is the relative electrical permittivity. The capacitance variation is then strictly related to a change in the thickness of the dielectric material or the metal plate’s surface area, and it varies linearly with the applied force or pressure. Using proper readout circuitry, it is possible to transduce this capacitance variation in a voltage output signal. Despite a good sensitivity, capacitive sensors show a limited detection range, mainly due to a decrease in the elasticity of the final device when metal electrodes are deposited on the dielectric elastomer. For this reason, in a wearable application, the conductive electrodes are usually made of elastic materials such as a mixture of polydimethylsiloxane (PDMS) or EcoFlex and silver nanowires (AgNWs) [55,61], liquid indium gallium arsenide (GaInAs) alloy paints [57], or conductive hydrogels [28].

The direct piezoelectric effect (Figure 6b-iii) allows transducing the mechanical energy caused by deformation or strain into electrical energy, usually collected as a voltage signal [84,85]. This phenomenon is based on the property of some ceramic crystals and polymeric materials to generate dynamic charge separation and a consequent electric potential due to reorientation of the dipoles inside the material lattice while subjected to a force or mechanical stress. Materials used to produce piezoelectric sensors can be organic, such as polyvinylidene fluoride (PVDF), polyacid(D-lactic) (PDLA), and polyacid(L-lactic) (PLLA), or inorganic such as aluminum nitride (AIN), lithium niobite (LiNbO_3_), barium titanate (BaTiO_3_), lead zirconate titanate (PZT), zinc oxide (ZnO), and quartz. Organic polymers are often preferred for their biocompatibility, simpler manufacture, and low cost. However, they show a lower piezoelectric response when compared to their inorganic counterpart [86]. One of the most used materials for piezoelectric wearable sensors is PVDF which has excellent physical properties, high polarization, and good flexibility, often combined with tetrafluoroethylene (TrFE) to improve the piezoelectric coefficients [87,88].

The triboelectric effect is a contact electrification phenomenon whereby a voltage signal is generated by transferring electric charges between two materials when they are in contact with each other and are subjected to friction and rubbing (Figure 6b-iv)—during the rubbing, one material gives electrons, charging itself positively. In contrast, the other material receives the released electrons, charging itself negatively as a consequence. Triboelectric-based sensors can be classified into four categories, according to their operating modalities: (i) vertical contact–separation sensors; (ii) in-plane sliding sensors; (iii) single-electrode sensors; (iv) freestanding triboelectric sensors [43,89,90,91]. Triboelectric sensors add the potential advantage of being self-powered since the output signal generated by the electrification process can be harvested and stored in batteries. For this reason, triboelectric sensors are widely employed in wearable applications, ensuring long-lasting operations such as joint-movement tracking and breathing monitoring [26]. Different materials can be used to fabricate triboelectric charge-generating and charge-trapping layers and electrodes. Usually, they are polymer-based, highly flexible materials, allowing the device to be embedded inside clothing or placed conformably in direct contact with the skin [92]. Poly(dimethylsiloxane) (PDMS), fluorinated ethylene propylene (FEP), fluorinated poly(ethylene terephthalate) (PET), and Poly(vinylidene fluoride-co-trifluoroethylene) (PVDF–TrFE) are some of the most used materials for the charge generating layer [93]. Polystyrene (PS), polyimide (PI), and PVDF, having many trapping sites due to the non-uniform energy levels along their main chains, are often used as charge-trapping layers. In some cases, they include nanoparticles, such as Ag nanowires (AgNWs), which promote trapping the electrostatically induced charges at the metal–dielectric interfaces. Solid and flexible electrodes, including metals, graphene, and indium tin oxide (ITO), are usually used for the charge-collecting layer together with conductive elastic nanocomposites, which have stable electrical properties in various mechanical environments.

A combination of the previously mentioned transduction methods is often developed to improve the response of the final hybrid sensor to the applied stimuli. Hybrid devices made by combining triboelectric and piezoresistive effects or triboelectric and piezoelectric effects are extensively explored [18,43]. A combination of inertial and piezoresistive transduction methods is also available, especially for monitoring joint movements [59], gait characteristics [63], or respiratory activity [94].

A summary of the electromechanical transduction technologies discussed above is reported in Table 3. Here a classification of the sensors and systems has been carried out, taking into account the transduction mechanism, the ability of the sensors to generate a signal without driving it by an external source (hereafter referred to as active/passive element), strengths, main drawbacks, and area of the body where the sensor can be applied.

**Figure 7:**
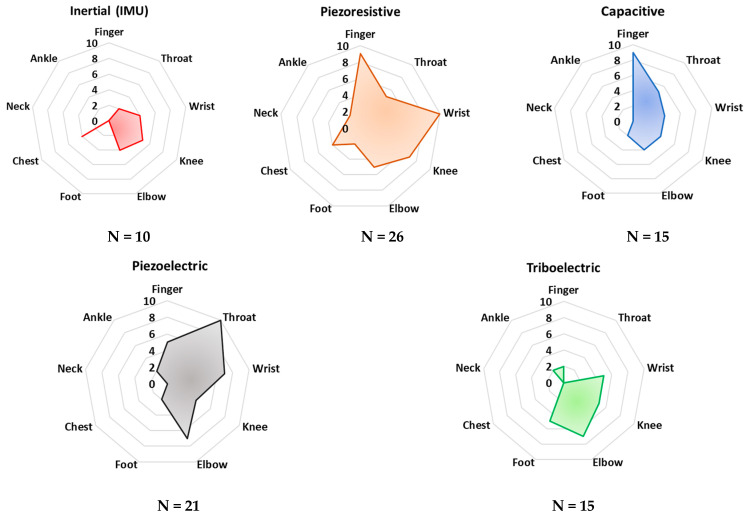
Radar charts for comparison of the main transduction mechanisms to monitor human bio–physical parameters according to the area of sensor application on the body. The scale (0–10) indicates the “frequency” with which a specific sensing technology is applied for monitoring a given body area. Additionally, the analyzed population (N) is reported for each sensing technology.

Establishing a comparison between the various technologies to better understand how they could work in post-operative rehabilitation and sports performance monitoring proved to be a complex task. To help the reader understand the complexity of the scenarios for these technologies, we present two summarizing figures. The radar charts of Figure 7 report a statistical analysis of the analyzed papers to determine the ability of that sensor/technology to detect frequency variations in the desired physiological activity, e.g., breathing, bending, walking, running frequency, and so on. Specifically, the “frequency” parameter was introduced for this purpose. It represents the number of analyzed papers (a total of 112 documents) where a given sensing technology was employed to monitor specific body areas (i.e., ankle, neck, chest, foot, etc.). The resulting paper numbers were normalized on a scale from 0 to 10, considering the range of the analyzed sensing technologies and the considered application areas. In addition, the analysis was carried out taking into account just the main application area on the body suggested by the authors of each paper.

**Figure 8:**
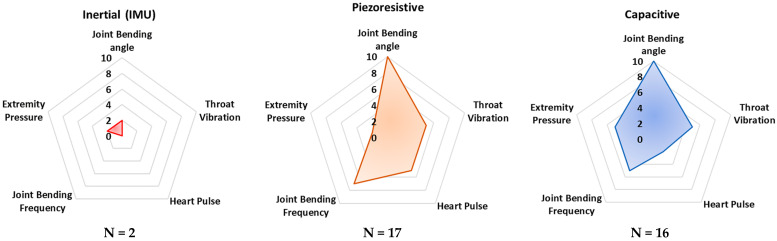

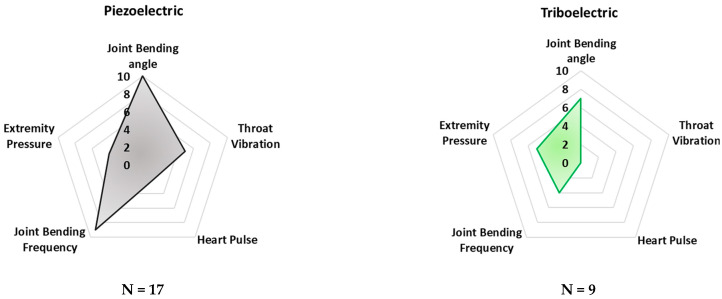
Radar chart comparing the considered transduction mechanisms according to the main detected biophysical quantities. The scale (0–10) indicates the “frequency” with which a specific sensing technology is applied for monitoring the specific parameter.

Additionally, the radar charts in Figure 8 depict the application rate of the technology/sensors analyzed in monitoring typical biophysical parameters, such as joint bending angles and frequency, extremity pressure, heart pulse, and throat vibration. Similarly, the “frequency parameter” was calculated to evaluate the propensity of a given sensing technology to detect the considered biophysical parameters. Similarly, in this case, only the main acquired parameter was selected for each analyzed paper.

These radar charts intend to provide a comparative graphical analysis of all the devices and sensors analyzed in this review paper. They were built considering the normalized number of cited research works whose developed technology—capacitive, triboelectric, piezoresistive, piezoelectric, and inertial—is applied to a specific body area and the desired parameter which can be detected and monitored.

As can be noted in Figure 7, piezoresistive and capacitive sensors are widely used for monitoring finger and wrist movements. Their employment can be justified by the availability of textile-based sensors, widely used for these applications since they feature a very low profile and high flexibility. However, both sensors’ typologies are passive and thus require the application of polarization, which is a drawback for low-power applications. Furthermore, piezoelectric sensors are commonly applied for detecting throat movements. Indeed, several examples of thin-film piezoelectric layers are proposed in the scientific literature, showing high sensitivity, flexibility, and biocompatibility, enabling discreet and safe detection of body movements.

In addition, from Figure 8 it is evident that piezoresistive, capacitive, and piezoelectric sensor technologies are currently dominant for detecting joint movements (angles and frequency), as well as throat vibrations, i.e., they are applied to voice recognition. Triboelectric sensors are commonly applied to detect pressures from different body parts—e.g., feet and hands. Nevertheless, this sensor technology is not mature enough to be applied to large-scale devices. In conclusion, from the reported analysis, inertial sensors are used to a lesser extent than the other sensor technologies, probably due to their high sensitivity to extraneous body motions, inducing motion artifacts.

## 4. Figures of Merit for Performance Comparison

Different characteristic parameters can be identified to compare and classify the working mechanisms used for implementing the wearable sensors and systems discussed in Section 3 and quantify their efficiency and performance. These parameters will be used as figures of merit (FoM) for carrying out comparative analysis of the sensing devices reported in Section 5.

One of the most important parameters is the sensitivity (S), defined as the ratio between the variation in the output signal dX and the related variation in the input dP:

(3) $$S=\frac{\mathrm{dX}}{\mathrm{dP}}$$

states that the input stimulus (compression or traction) generates a significant output variation in a sensor with high sensitivity [101]. However, in some cases, it is useful to resort to the concept of normalized sensitivity to take into account in this parameter the sensor’s resting value (X_0_) [102]:

(4) $$S=\frac{∆{X/X}_{0}}{\mathrm{dP}}$$

A second parameter useful to describe the performance, especially of strain piezoresistive sensors, is the gauge factor G_f_ defined as

(5) $$G_{f}=\frac{∆R/R_{0}}{∆L/L_{0}}$$

which is the ratio between the normalized resistance variation ΔR compared to the resting resistance R_0_ as a function of the elongation variation ΔL of the resistive stripe compared to the resting length L_0_. Typical values of this parameter range between 2 and >100, depending on the shape and type of material comprising the stain sensor [103].

Extensibility E, also called Young’s modulus, is an intrinsic feature of the material used. It defines the maximal threshold beyond which the ratio between the strain and the traction applied to the material causes the sensor to lose sensitivity and linear behavior. The Young’s modulus is calculated according to the following equation:

(6) $$E=\;\frac{d\sigma }{d\varepsilon }$$

where ε is the strain generated when a traction (or stress) σ is applied. A Young’s modulus approaching the elastic properties of the skin is highly desirable, especially for wearable devices in direct contact with soft tissues [104].

Additional parameters are evaluated to define the performance of wearable sensors. The range between the minimum and maximum values the wearable sensor can detect is defined as the range of detection (RoD); beyond the maximum value, the sensor is no longer sensitive to input variations [105]. The sensor response time is the time interval between the application of the external stimulus and the generation of the transduced output signal (a critical factor in dynamic and real-time activity tracking) [106].

Linearity is the percentage of deviation of the measured signal from the regression line and allows for evaluation of the signal stability in specific applications [105]. Among the other FoM, it is certainly worth mentioning the precision, which indicates the capability of the sensor to keep the measurements reproducible. Another fundamental FoM is the resolution intended as the lowest observable incremental change of an input parameter. Resolution can be represented in absolute terms or as a percentage of the reading (or the full-scale reading). Furthermore, the greatest deviation between the sensor measurement against the reference obtained by the main or a good reliable standard is defined as accuracy [105]. Furthermore, the offset error is the deviance in the sensor output from a reference value at the detection range’s bottom end. Lastly, power consumption is critical in wearable applications, especially for long monitoring of daily life.

## 5. Overview of Available Wearable Technologies for Body Motion Monitoring

Wearable devices for monitoring body part movements and physiological parameters improve standard and traditional rehabilitation therapies since they can provide important feedback to the users—both patients and therapists—additionally allowing to customize the training path of the athletes. Section 5 reports an overview of the most recent and innovative wearable sensors and systems based on the transduction technologies detailed in Section 3 and suitable both for patient rehabilitation after injuries or surgeries and for tracking the activities of athletes.

### 5.1. Devices and Systems for Post-Operative Rehabilitation

Patients’ joint movements are often monitored with two useful technologies: inertial sensors (IMU) and vision-based sensors (e.g., Microsoft Kinect) [20,97,99,107,108]. Ianuculescu et al. proposed a device able to provide post-operative tracking of patient rehabilitation from home, achieving the same therapy results reached in a hospitalized structure [54]. The re.flex system consists of two IMU sensors equipped with a three-axis gyroscope and an accelerometer for tracking knee movements. A proper software application for mobile devices was also developed to acquire and process the acquired data from the IMU sensors. Combining the data collected by the accelerometers and the gyroscopes, the system can measure up to twelve parameters, such as speed, gravity, and three-dimensional orientation, allowing the user to monitor movements during exercise training.

Additionally, the mobile application allows the user to interface with the measured data; the patient and the physician can visualize the movements of the leg through a real-time three-dimensional avatar and follow the exercises selected and customized by the physiotherapist. A self-calibrating algorithm automatically detects the sensor’s position and calibrates it accordingly, to achieve the best output response. This automatic calibration allows the user to easily attach the sensors to the injured area. The system was tested for monitoring the reconstruction of the anterior cruciate ligament of 30 patients. They were randomly separated into two groups 48 h after the surgery: the first group followed rehabilitation in a clinic, and the second group stayed at home using the re.flex monitoring system. For both groups, the treatment lasted three months. The group treated remotely with the re.flex system achieved an average Oxford knee score—an index that measures knee pain and function after reconstruction surgery—of 1.3 points higher than the group treated in the clinic.

**Figure 9:**
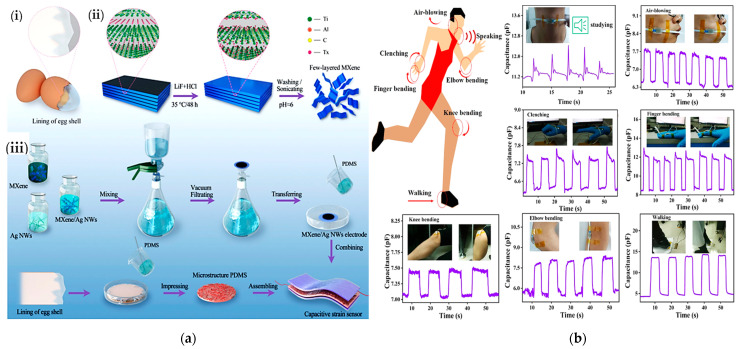

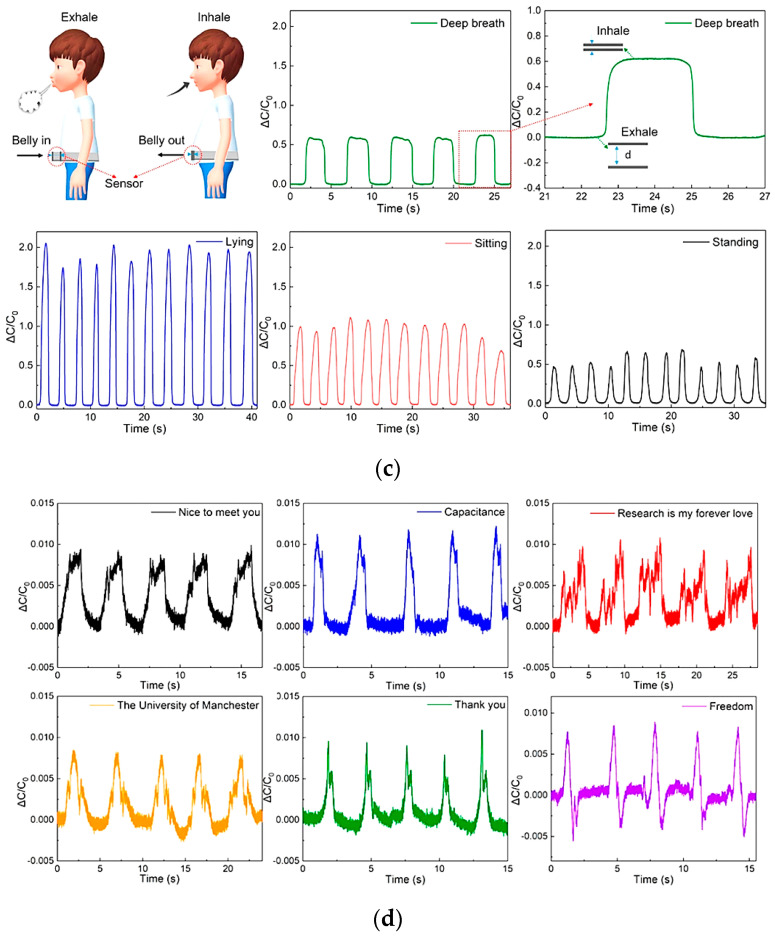
A capacitive pressure sensor’s construction (**a**). Illustration of the ESIM’s (eggshell inner membrane) structural elements (**i**); schematic showing the construction of MXene sheets (**ii**); and the manufacture of flexible capacitive pressure sensors (**iii**). Schematic sensor diagram detecting human physiological signals and body motions. From top left to bottom right: vocal cord vibration signal when pronouncing the word “studying”; capacitance responses from the repeated air blowing, clenching and releasing of finger/knee/elbow bending, and walking (**b**) [29]. Sensor output during exhalation and inhalation acquired as capacitance variation during deep breathing, measured in different positions: standing, sitting, and lying (**c**). Variation in the capacity during the slight vibrations of the vocal cords allowed to recognize “Nice to meet you”, “Capacitance”, “Research is my forever love”, “The University of Manchester”, “Thank you”, and “Freedom” (**d**) [53].

A capacitive sensor with a bio-inspired eggshell microstructure was proposed by He et al. for monitoring walking, air blowing, finger, knee, and elbow movements, and performing voice recognition [29]. The sensor was made of a thin dielectric layer with microstructured PDMS geometries embedded between two electrodes of MXene titanium carbide (Ti_3_C_2_Tx) mixed with Ag nanowires (Figure 9a). The sensor showed excellent sensitivity (i.e., 10.13 MPa^–1^) and a good response time (~ms) within a pressure range from a few kPa up to 600 kPa (Figure 9b).

Chen et al. developed a capacitive sensor with a textile structure, excellent conductivity and elasticity, and high comfortability when worn [53]. The sensor was fabricated by coating the starting fabric with nickel. A properly shaped fabric, with a size of 13 mm × 37 mm, was applied to the patient’s belly to evaluate breathing during rehabilitation exercises. One of the two electrodes was inserted inside the tights, the other into the inner part of a belt (Figure 9c).

By inhaling and exhaling, the patient expands and contracts the abdomen, varying the distance between the two electrodes and thus generating a capacitance variation (as shown in Figure 9d). Slight vocal cord vibrations were also monitored to distinguish sentences pronounced by the patient to diagnose and prevent dysphasia issues. After repeated bending cycles, a stress test was performed to verify the signal stability. After hundreds of bending cycles, the device’s capacity increased from 24.3 to 26 pF, showing good signal stability even after running a high number of cycles. Similarly, in [109], the authors presented novel Ag–NW-based capacitive strain sensors to sense skin deformations for tracking finger movements. The sensors’ characteristics were a high sensitivity (gauge factor ≈ 1), low Young’s modulus (96 kPa), linear response, high stretchability (150%), negligible hysteresis, and broad strain sensing range greater than required to detect human movements. The experimental results demonstrated that the skin deformations acquired with the developed capacitive sensors are highly correlated with those obtained with an eight-camera optical tracking system. The authors suggest using these sensors to monitor hand movements in patients affected by chronic diseases such as cerebral palsy, spinal cord injury, or muscular dystrophy.

Other hand-worn systems were developed to assist users in rehabilitation from stroke and traumatic brain injury [110]. In Ref. [111], the authors presented an innovative robotic system for assisting patients with repetitive hand exercises. Specifically, they created a wearable therapy device driven by pneumatic muscles (PM) that delivers the supportive forces needed for gripping and finger extension. The robot fingers have two independent degrees of freedom (DoF); also, position and force data are acquired by integrated sensors for controlling the robot and evaluating its performance. A fuzzy PID controller was developed to control the robot’s movements, which, as demonstrated by experimental tests, performs better than the standard PID controller.

In Ref. [112], the authors presented a hand rehabilitation system that aids patients in performing repetitive training. It comprises a Myo armband, a robotic glove, a Unity3D, and a videogame processing platform. The Myo armband gathers spatial and gestural data using integrated IMU and EMG sensors. The acquired data are wirelessly transmitted to Unity3D, which derives the controls for the robotic hand. This last device includes two stepper motors for controlling the opening/closing of the hand. In a hand-training game created in Unity 3D, players must pick up, hold, move, and drop a cube in progressively challenging puzzle stages. To make the user feel more engaged in the game, the user sees virtual hands and arms that mimic their actions.

Furthermore, with 11 elastic actuators that impart extension torques to the finger and thumb joints, the HandSOME II device helps the patient expand their hand [113]. Despite the device’s DoFs not being aligned with those of human joints, a new mechanical design delivers forces orthogonally to the finger segments. Experimental tests were carried out on seven post-stroke patients affected by hand disabilities, demonstrating that HandSOME II can enhance the extension angles and range of motion in all finger joints.

Innovative materials are continuously developed for implementing sensors to detect body movements and physiological parameters, which can easily be integrated into the clothes. Tan et al. developed a textile-based piezoresistive sole to monitor the patient’s walking motion during rehabilitation and prevent diseases such as diabetic ulcers and foot deformation [62]. The sensor was made with a cotton electrode coated with reduced graphene oxide (rGO) and a fabric electrode with a silver (Ag) circuit inside. Six sensors were inserted into the shoe sole, allowing for real-time observation of the foot pressure distribution during movement, achieving an accurate pressure map. The sensors were connected to a small electronic board equipped with an acquisition circuit, a Bluetooth module for wireless data transmission, and a battery for power supply. The sensor had a 170 ms response time, a 3.90 kPa^–1^ sensitivity in a pressure range between 0 and 36 kPa, and exhibited good stability during a durability test of up to 1000 cycles.

Weak stimuli such as breathing, swallowing, chewing, finger flexion, muscle vibration, and joint movements were successfully measured by exploiting a piezoelectric sensor developed by Wang et al. [21]. The sensor was made of poly(vinylidene fluoride–trifluoro ethylene) multi-walled carbon nanotubes (p(VDF–TrFE)/MWCNTs) grown in their β-phase and sandwiched between two gold electrodes. A transparent thin silicone film covered the device and protected it without altering the final flexibility. The sensor’s sensitivity and extensibility were calculated, resulting in 540 mV/N and 0.986 Gpa, respectively. Studies on adult patients were carried out, placing the sensor in contact with the skin on the wrist and acquiring the heartbeat. A measurement of 84 beats per minute—corresponding to a standard heartbeat—was recorded even with a very weak input stimulus. The breathing frequency was also determined with a peak voltage of 0.1 V, as well as the swallowing, by applying the sensor to the throat. A peak voltage of 0.75 V was easily detected, allowing measuring also the time the muscles take to complete the whole swallowing movement. Chewing gestures were finally measured. Here signal peaks of 0.3 V amplitude were detected. Finger bending and bending of the wrist were also detected. Figure 6a shows the plot of the acquired signal during the previously mentioned tests. Kim et al. proposed an innovative sensor to measure gait by exploiting the piezoelectric effect [24].

**Figure 10:**
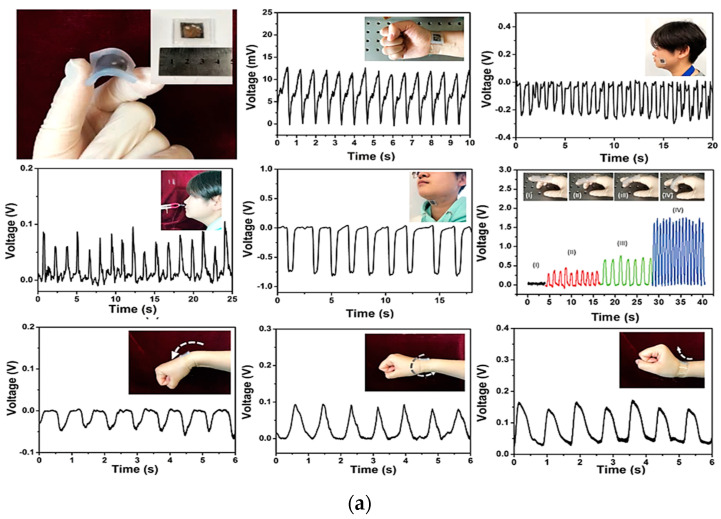

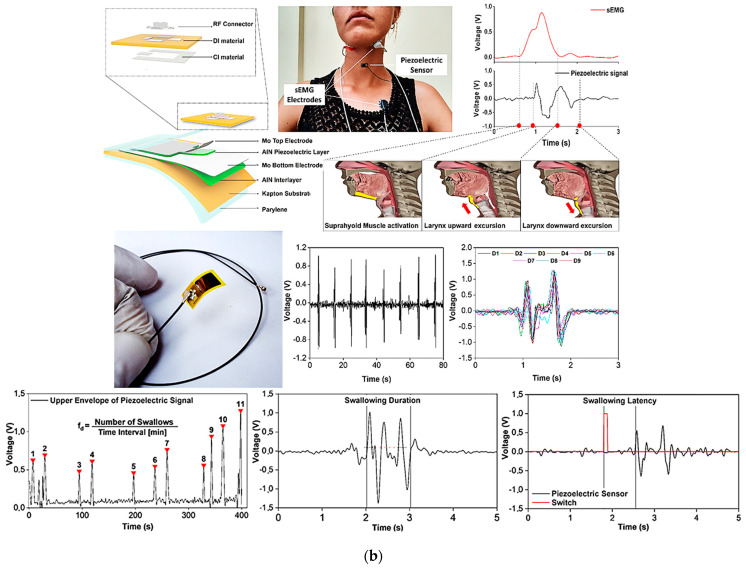
PVDF flexible sensor and the corresponding output voltage over time, and position for the detection of heartbeat, breathing, swallowing, and chewing. Additionally, maximum voltage values depending on the flexion levels of the finger, upper bending, lower bending, and wrist rotation are also reported (**a**) [21]. Flexible piezoelectric sensor with the corresponding exploded drawing showing the multilayered structure (**b**). Here the deglutition wave recognition and segmentation with the comparison of the sEMG recorded signals of a single swallow action are reported. Additionally, spontaneous frequency, duration time, and latency of a swallowing act are also displayed [34].

The sensor, made of a piezoelectric PVDF layer, was fixed on one side by two rubber bands, while on the opposite side was attached a polyester thread where the mechanical tension was applied (Figure 10a). A flexible printed circuit board was used to acquire the output voltage by connecting two electrodes to the upper and lower surfaces of the PVDF film with carbon-based conductive tape. The upper surface’s ends of the PVDF strip were protected by a polyethylene terephthalate (PET) layer. Only one of the lower surfaces was fixed to the PET substrate, allowing the PVDF strip to flow freely over the substrate when a traction stimulus was applied to the free side. Kapton tape was used to electrically insulate the sensor. The peak-to-peak open circuit voltage was measured as a function of the upper limb movement; seven sensors were then embedded in commercial stockings, and medical bands were applied to the wrist, elbow, chest, and shoulder.

A minimally invasive sensor for monitoring of the deglutition capabilities of the patients to evaluate dysphagia issues was developed by Natta et al. [34]. A piezoelectric sensor worn directly on the throat was used to transduce larynx movements. The sensor was made using a piezoelectric aluminum nitride (AlN) thin film, sandwiched between two molybdenum (Mo) electrodes deposited on a very thin flexible polyimide substrate, reaching an overall thickness of 26 µm and a weight <2 g. A sticky PDMS–PEIE polymer layer was then used to attach the sensor to the skin (Figure 10b). The output voltage was successfully transmitted to a smartphone via wireless Bluetooth technology after being amplified and filtered by a suitable conditioning circuit. Duration of the swallowing act, frequency of spontaneous saliva deglutition, and latency were easily detected by this sensor, allowing an objective evaluation of the subject’s swallowing capability and providing, at the same time, an early diagnosis of pathological conditions. The data collected were compared with standard surface electromyography (sEMG) signals, showing that the sensor is a valid alternative to classical devices (Figure 10b).

A sensor to detect and monitor swallowing by converting tongue movements into an output voltage generated by the triboelectric effect in single-electrode mode, also exploited to self-power the sensor itself thereby significantly reducing the power consumption of the whole system, was developed by Yun et al. [33].

Three layers of different materials were used for fabricating the triboelectric-nanogenerator for swallowing rehabilitation (TSR): a layer of polytetrafluoroethylene (PTFE) for triboelectric induction, a copper (Cu) electrode, and a top layer of polyethylene terephthalate (PET). The triboelectric nanogenerator for swallowing rehabilitation was then applied to the palate to detect tongue movements. It demonstrated good sensitivity of 47 mV/kPa even to weak tongue pressures ranging from 20 to 100 kPa. Two different TSR devices were then characterized, the TSR–B with bare PTFE and the TSR–T60 with 60° tilted PTFE. Peaks of the open-circuit voltage of approximately 13.10 V (with a short-circuit current of 0.512A) were measured for the TSR–B. An increment of the output voltage of approximately 189%—approximately 24.76 V—was observed for the tilted TSR–T60. The power density was then calculated to define the energy generation for self-power low-consumption devices when the sensors were connected to a variable resistance load.

**Figure 11:**
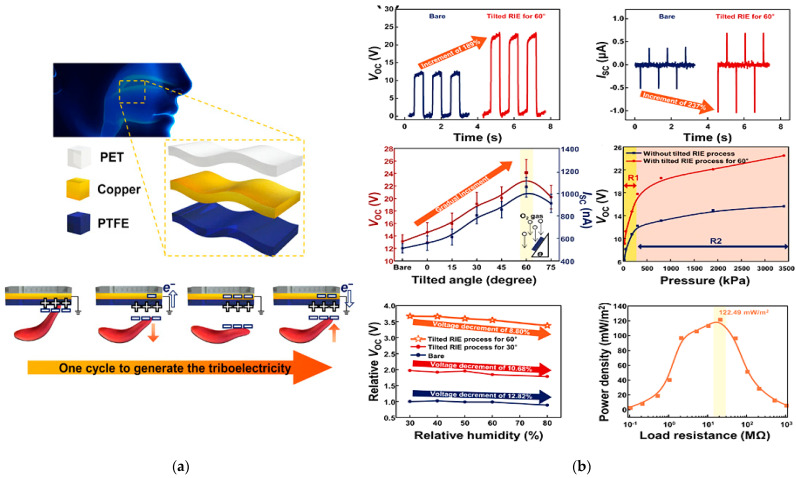
Schematic illustration of fabricated TSR and the working mechanism in one operating cycle (**a**). Open circuit voltage and short circuit current measured on TSR–B and TSR–T60, including the tension produced when the tilting varies, the pressure sensitivity of both the sensors, the output voltage when the humidity increases up to 80%, and the power density according to the load resistors (**b**) [33].

Additionally, the sensors’ sensitivity was calculated by analyzing the output voltage curve as a function of the applied pressure. In this case, the pressure range was split into two sub-ranges, namely R1 from 20 to 180 kPa and R2 from 300 to 3.4 MPa. The voltage vs. pressure curve slope was then 35 mV/kPA for the TSR–B and 47 mV/kPa for the TSR–T60 in the range R1 and 1.10 mV/kPa and 2.20 mV/kPa in the range R2, respectively. Finally, humidity tests were also performed, increasing the humidity percentage from 30% to 80% to characterize the sensor in a humid environment, reproducing similar test conditions to the mouth. Figure 11 displays a schematic illustration of the triboelectric-based sensor following one cycle of tongue movement, the generated output response, the sensitivity, and its performance when changing the humidity and environmental conditions.

Besides post-operative rehabilitation after trauma or injuries, numerous wearable devices were presented in the scientific literature for rehabilitation after damage to the nervous system due to stroke. In particular, G. Li et al. presented an untethered adaptable thumb exoskeleton that actively supports the thumb’s three degrees of freedom of motion [114]. An adaptive thumb and spherical mechanisms constitute the exoskeleton, including sliders made of ball-type linear bearings to reduce the rolling friction. The exoskeleton’s workspace, self-alignment, interface forces, admission controller, and grasping aid performance were tested experimentally. The workspace and self-aligning tests indicated that the exoskeleton could obtain a significant thumb joint workspace and self-alignment. The test results suggested that the presented robot-assisted rehabilitation system could reduce the tangential interaction force by 76.8% and enhance comfort. Additionally, experiments demonstrated that the exoskeleton could improve the thumb’s grasping capability.

Similarly, in [115], the authors used wearable inertial sensors placed on different body parts, which gather the movement data and transmit them through Zigbee to a PC. Pre-processing methods were applied to enhance the signal quality before extracting movement features through the multi-sensor fusion technique. Then, the DTW (dynamic time warping) method was employed to determine movement scores. These scores are provided as input to a machine learning model (SVR—support vector regression), using the FMA (Fungl–Meyer assessment) indexes as the output classes’ labels. The trial research involved 120 stroke patients, and statistical evaluations of the two assessment techniques were conducted. The experimental results revealed that R^2^ for the score regression analysis for both approaches was 0.9667, whereas the mean deviation was 0.30., The percentage of deviations and relative deviations inside the interval mean ± 1.96 SD (standard deviation) were equal to 92.50% and 95.83%. Additionally, Y. Ren et al. proposed a robotic ankle device to generate extensive passive and active movement training in post-stroke patients [116]. It includes a force sensor, a rotational actuator, a leg brace, and a foot holder. Adjusting the straps may accommodate each person’s leg and foot size. The device is actuated by a DC motor controlled by an encoder to measure the angle change. The resulting system guides the patients in motor relearning, providing motivational feedback to the patient during the rehabilitation training in the active training modality. Experimental tests on ten post-stroke patients demonstrated that 12 sessions could improve motor control ability and neuroplasticity.

Table 4 outlines some of the most important specifications of the analyzed sensors and devices so far, performing a classification according to sensing technology, used materials, sensitivity, transmission technology, area of the body where the sensor is usually applied, response time, and operating range.

### 5.2. Devices and Systems for Tracking an Athlete’s Performance

The current wearable sensors for sports tracking still have several limits to overcome, such as the high cost and high energy consumption, as well as poor fitting features and elasticity making them uncomfortable to be worn and hamper the movements during sports activities. However, innovative and challenging solutions have recently been developed in different sports fields, such as combat sports, basketball, volleyball, soccer, running, and weightlifting [117,118,119,120,121,122,123,124].

Masihi et al. designed a capacitive pressure sensor to detect impacts on the player’s head while using a helmet [46]. The transducer consists of two conductive textile electrodes and a porous layer of PDMS as a dielectric layer. The porosity of the dielectric layer was obtained by dissolving carbon dioxide (CO_2_) gas in a compound of PDMS and sodium bicarbonate (NaHCO_3_) and mixing it with nitric acid (HNO_3_). With this mixture, nine sensors with different porosities and dielectric constants were fabricated. The difference in the dielectric constant is mainly due to the percentage of HNO_3_ used. From laboratory tests, the device with 15:1 as the PDMS/HNO_3_ ratio had the highest sensitivity of 0.3 kPa^–1^ for applied pressures <50 Pa and 3.2 MPa^–1^ in the pressure range from 0.2 to 1 MPa.

**Figure 12:**
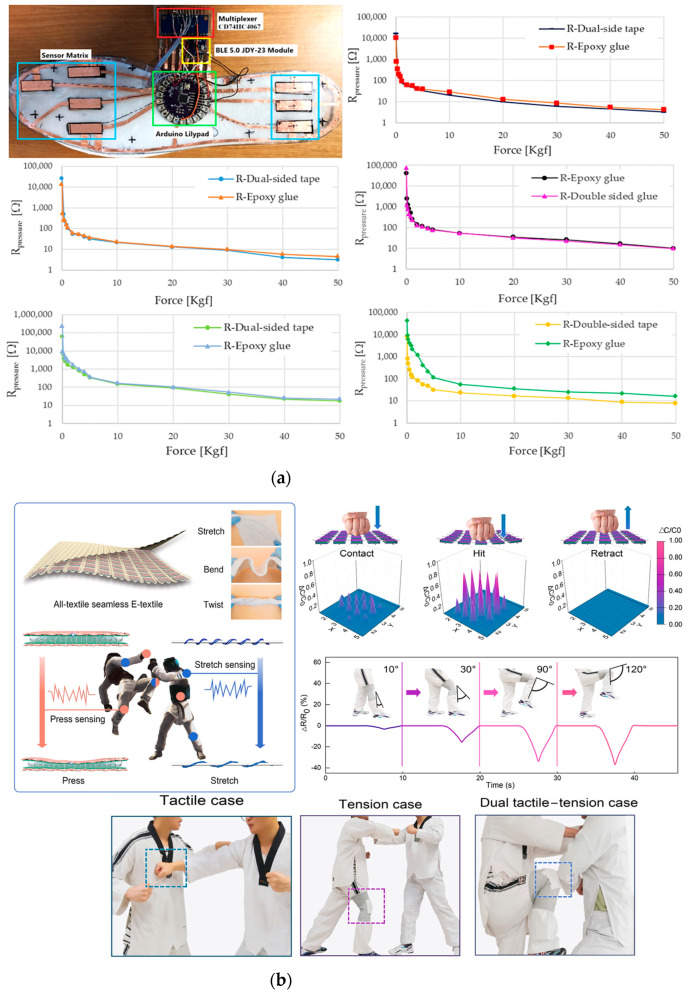
Examples of capacitive and piezoresistive hybrid sensors: (**a**) The developed sole with the piezoelectric integrated sensors, the acquisition system, and the Bluetooth module. Below, the characteristic curves R vs. F (in Kgf and with a logarithmic scale) for five Velostat-based pressure sensors are shown for different sizes, 3 cm × 3 cm, 1 cm × 1 cm, and 3 cm × 1 cm [63]. The yarn matrix structure in the inset shows some examples of the final fabric’s elongation, bending, and twisting (**b**). A map of the pressure distribution during the hit cycle and resistance variation at different knee bending angles are shown on the right. Some practical scenarios of using the integrated sensor on the chest and knee are also reported [40].

De Fazio et al. developed a self-powered smart insole based on piezoresistive and inertial sensors (Figure 12a). The insole relies on a matrix of eight custom piezoresistive sensors developed using a pressure-sensitive polymeric layer (i.e., Velostat), whose resistive values are read by an acquisition system made with a multiplexer and Arduino Lilypad [63]. The insole is also equipped with an inertial sensor to monitor walking and step counting, a power module with a battery, and a piezoelectric harvester to recharge it, exploiting the same pressures applied during gait and walking movements. A Bluetooth module is used to transmit the data to a PC. The insole was tested while walking at a speed of 1 m/s, and the acquired data were used to build a distribution pressure map.

A hybrid sensor used to monitor the technical movements of taekwondo athletes was made by Ma et al. The proposed e-textile sensor can detect elongation and pressure stimuli by exploiting both piezoresistive and capacitive effects [40]. The sensor was fabricated starting from commercial polyacrylonitrile (PAN) fibers, coated with carbon nanotubes (CNTs), and reduced graphene oxide (rGO) to make it conductive. Then, the two achieved fibers were rolled up, forming a helical structure wound around a polyurethane (PU) multifilament, obtaining a conductive core-sheath yarn sensitive to stretch (Figure 12b).

The sensor was designed considering the dobok taekwondo uniform should withstand substantial pressure impacts (>100 kPa) and extensive stretch movements (with a strain > 50%). Core-sheath yarns were fabricated with an increasing winding number from 20 to 70 along a distance of 10 cm. To test the elongation ability of the e-textile when used as a strain sensor, it was stretched, and the relative resistance variation ΔR/R_0_ as a function of the stretching was measured. The characterization results showed that yarns from 20 to 50 twists exhibited a negative variation in the resistance response, while yarns with 60 and 70 twists positively increased. Experiments were also performed to characterize the capacitive behavior as a pressure sensor by acquiring the relative capacity variation ΔC/C_0_ in a range of pressures up to 110 kPa (a typical practical case). In the ranges 0–9 kPa, 9–37 kPa, and 37–110 kPa the sensitivity was then calculated, and values of 0.112 kPa^–1^, 0.0283 kPa^–1^, and 0.0021 kPa^–1^, respectively, were reported. The sensor also had a fast response to pressure pulses between 1.5 and 10 kPa and good stability of the output response even after 100,000 cycles of long tests. Moreover, practical cases were successfully analyzed, and the sensor was integrated into the uniform on the chest to identify the pressure distribution of a blow and on the knee to monitor the athlete’s kicks, allowing to identify the angle of the bending knee (the experimental results are reported in Figure 12b).

**Figure 13:**
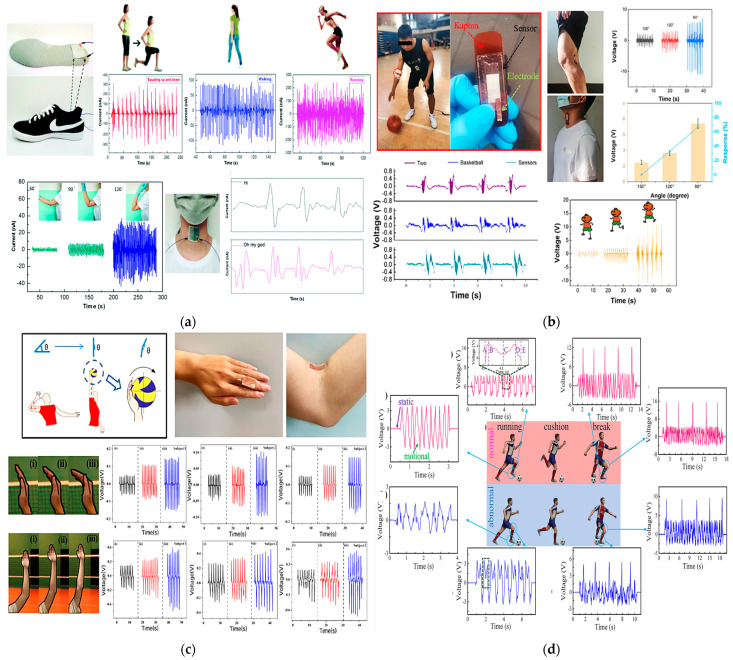
Piezoelectric sensors: PVDF/BaTiO_3_-based sensor integrated into a sole with the corresponding output currents generated during squatting, walking, and running activities, elbow extension and flexion to 60°, 90°, and 120°, and the pronunciation of short sentences (**a**) [22]. PVDF/DMF-based sensor applied on an athlete’s elbow and the corresponding voltage signals generated at different bending angles and during different physical activities (**b**) [39]. A schematic diagram of different bending angles of the palm during the test of the PVDF sensor. The output piezoelectric voltages of three subjects when finger and elbow bending angle change are also reported (**c**) [42]. Soccer player motion monitoring test using the PVDF–HFP-based sensor (**d**). Pictures of a soccer players’ actions, showing normal ankle motion, abnormal ankle motion, normal knee motion, and abnormal knee motion. Moreover, the “brake” action of the same motion is also monitored and reported [64].

A piezoelectric pressure sensor for a wireless wearable sensing system obtained from the combination of tetragonal-phase BaTiO_3_ nanowires and electrospun PVDF nanofibers was designed and proposed by Guo et al. [22]. The system also includes an electronic board with a module for amplifying the acquired signal, a power converter, a module for controlling the data conversion, and a Bluetooth module for wireless transmission. The output voltage was evaluated using an ad hoc set-up and applying a controlled pressure. The maximum output current value measured on 100% PVDF fibers was approximately 47 nA, while that measured on the PVDF nanocomposite with 3% BaTiO_3_ nanowires was 105 nA. The sensor exhibits a sensitivity of 0.017 kPa^–1^ and a response time of 290 ms, estimated from the voltage-over-time curve in one contact cycle. A durability test of 1750 contact cycles at a frequency of 3.5 Hz was also performed, showing the sensor has very good signal stability over time. Practical tests were carried out. The sensor was then embedded inside a sole to track walking, running, and squatting movements. Current peaks of 97 nA, 241 nA, and 331 nA were measured, respectively. The sensor was also attached to the elbow, evaluating the flexion and extension angles at a frequency of 1.15 Hz. Here, the maximum current generated was 36 nA at an elbow bending angle of 120°. The sensor was finally used to detect slight vibrations of the vocal cords when placed when pronouncing short sentences, such as “Hi” and “Oh my God”. The acquired signal during these tests is reported in Figure 13a.

Still exploiting the piezoelectric effect, Zhao et al. designed a self-powered sensor to monitor basketball players’ movements and gestures [39]. The sensor was fabricated starting from a mixture of dimethyl formaldehyde (DMF) and PVDF poured onto a silicone substrate. Then, thin Ag electrodes of 300 nm thickness were applied on both sides of the piezoelectric film. After wiring it, the sensor was attached to the elbow. Generated voltage spikes of 2.172 V at an angle of 150°, 3.48 V at 120°, 6.052 V at 90°, and 8.08 V at 60° were measured. Moreover, the relative piezoelectric output at different frequencies was also acquired, keeping the same bending angle. Voltages of 5.24 V, 5.24 V, 5.328 V, and 5.32 V were measured at 0.5, 1, 1.5, and 2 Hz, respectively. Finally, the sensor was positioned on the popliteal fossa of an athlete for in vivo tests; the generated voltage during the jumping, walking, and running activities was successfully acquired, showing voltage peaks of 9.387 V, 1.02 V, and 2.04 V, respectively (Figure 13b).

Liu et al. proposed a similar self-powered piezoelectric sensor composed of polarized PVDF film for voice recognition and monitoring of arm and hand movements of volleyball players. Here, the sensor was also used as an energy harvester to scavenge voltage from the continuous movements of the volleyball player to charge a capacitor and power up a Bluetooth module for wireless transmissions [42]. The signal generated by the sensor during hand bending and movements is displayed in Figure 13c.

In addition, Li et al. developed a pressure sensor that, thanks to its high sensitivity (approximately 1.9 V/kPa^–1^), was used to detect very slight athlete movements and prevent overtraining injuries [64]. The sensor was made of polyvinylidene fluoride and hexafluoropropylene (PVDF–HFP) nanofibers, obtained through electrospinning and painted with zinc oxide (ZnO) solution using an atomizing gun. The piezoelectric layer obtained was sandwiched between two aluminum layers. Wires were then used to collect the voltage generated by the sensor. Different amplitude forces—from 0.02 N up to 0.5 N—at a frequency of 1 Hz were applied, and the corresponding response time was estimated at approximately 20 ms. Furthermore, the sensor’s sensitivity was calculated, with a maximum value of 1.92 V/kPa^–1^. Activities such as running and breaking, usually performed continuously during a match, were monitored (Figure 13d). 

**Figure 14:**
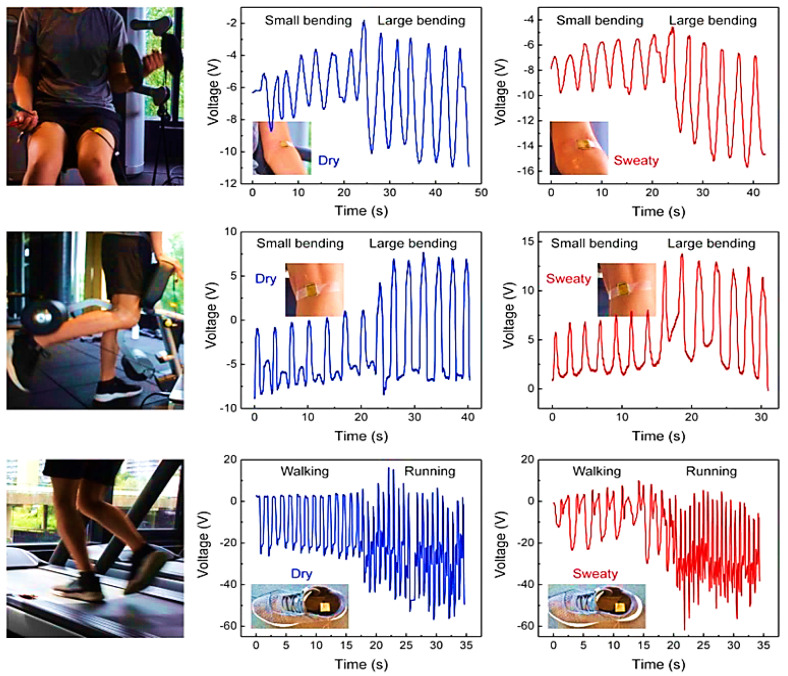
BSRW–TENG sensor applied to the elbow for monitoring biceps curl, leg curl, running, and walking—before and after sweating—with the corresponding plots of generated output voltages [41].

Li et al. [41] developed a low-cost, sweat-resistant triboelectric (BSRW–TENG) sensor for tracking movements during individual exercises such as leg and bicep curls and running. The sensor was used to monitor and track three different sports activities, including tests before and after sweating, to verify the waterproofing ability of the materials used and the stability of the output response. In the first case, the sensor was applied to the elbow to monitor the degree of bending during the biceps curl, obtaining feedback on the correct execution of the exercise. Similarly, the sensor was fixed on the back of the knee to monitor the leg curl. Finally, it was embedded into the sole of the shoes to detect the steps during walking and running. The acquired signals are displayed in Figure 14.

Table 5 outlines the most important specifications of the analyzed systems for tracking sports activities, classified according to sensing technology, used materials, sensitivity, transmission technology, area of the body where the sensor is usually applied, and response time and transmission technology used.

**Figure 15:**
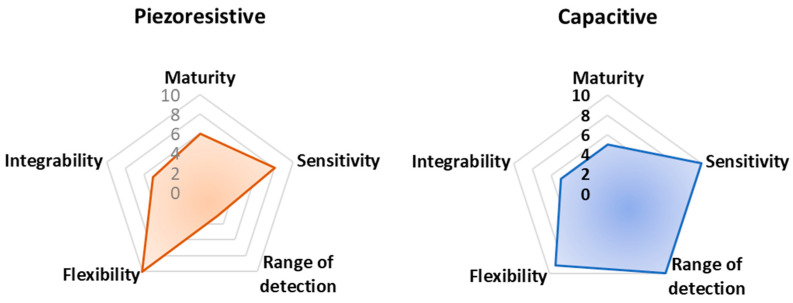

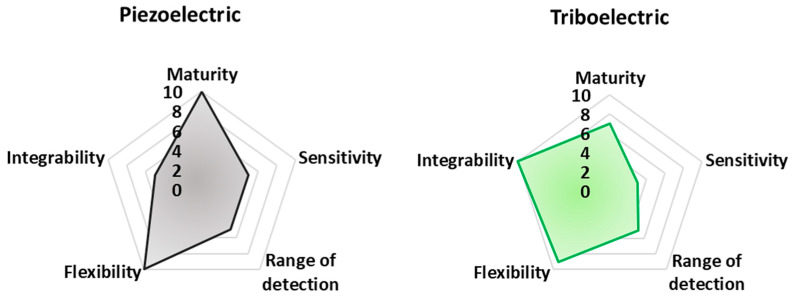
Radar chart showing a comparison between the analyzed sensors and devices according to their technological maturity, flexibility, ability for full integration into clothes and garments, sensitivity, and range of detection.

In Figure 15, a radar chart is reported, comparing the analyzed sensors and devices, both for rehabilitation and sports tracking purposes, according to their technological maturity, flexibility, ability to be completely integrated into clothes and garments, sensitivity, and range of detection.

The reported Figure 15 integrates evaluation criteria that are difficult to objectively estimate, referring to miscellaneous and very different properties for each analyzed technology. In particular, flexibility is evaluated as the maximum percentage of the analyzed work proposing fully flexible devices. Integrability is, instead, derived considering the number of analyzed works proposing a technology fully integrated or potentially integrable. Here, 10 (the maximum) is assigned to devices already integrated, whereas 5 and 0 are used for devices potentially integrable and not integrated at all, respectively.

Finally, technological maturity is determined considering the number of works/papers proposing that transduction mechanism so that the maximum number of cited works corresponds to 10 in the chart of technological maturity. These criteria have been qualitatively graded based on the authors’ judgment following a thorough review of the recent available literature. They represent a dynamic picture of the current state-of-the-art related to the analyzed applications. Despite being qualitative, the authors think the set of criteria is important for the scientific community to evaluate the advantages and disadvantages of each technology. Therefore, larger-surfaced hexagons should, in theory, represent the best transduction mechanisms to be utilized.

Finally, from these radar graphs, it is clear that piezoelectric sensor technology is the most mature, considering the numerous advances made in recent years from the point of view of performances (i.e., sensitivity, stability, robustness), integrability, manufacturing process, and costs. Additionally, piezoresistive and capacitive sensors offer good performance in terms of sensitivity; thanks to textile-based and polymeric solutions, such sensors enable their simple integration into cloths. However, piezoresistive sensors have a relatively reduced detection range, limiting the applicability of this sensor typology for detecting large movements. Triboelectric sensors allow high flexibility and integrability thanks to the multitude of materials that can be used for realizing such devices, including polymeric materials.

### 5.3. Overview of Datasets Related to Sport and Rehabilitation Applications

In the last few decades, the scientific community and companies have created several datasets containing heterogeneous signal parameters (e.g., accelerations, angles, pressures, etc.) that were acquired through different approaches (e.g., inertial, optical, piezoresistive, video, etc.), making them publicly available to researchers for developing future applications for rehabilitation and sports performance monitoring.

Considering the datasets gathered using camera-based and optical systems, the UI–PRMD (University of Idaho–Physical Rehabilitation Movement Data Set) collects motions associated with typical exercises performed by patients in physical therapy and rehabilitation programs [125]. The UI–PRMD is a freely accessible dataset, including ten rehabilitation movements; ten healthy people performed each motion ten times in front of Vicon optical trackers and Kinect cameras. The acquired data are the locations and angular orientations of the body joints in the skeletal models measured using both MoCap (motion capturing) systems. Likewise, the KIMORE (KInematic assessment of MOvement and clinical scores for remote monitoring of physical REhabilitation) dataset is a free dataset containing different rehabilitation exercises collected by an RGB–D sensor [126]. The dataset comprises skeletal data from five workouts performed by seventy-eight participants; these data are a time series of skeleton joint locations recovered from images taken with a Kinect. Furthermore, in [127], a multimodal dataset was proposed to analyze and measure the quality of movements carried out during karate moves. In particular, an optical motion capture system, consisting of nine cameras, was employed to record the user motions gathered from seven participants. The data underwent post-processing, which included identifying the markers, creating the models, and removing noise caused by “ghost” or jitter markers. The authors used the created dataset to assess the quality of karate moves.

Additionally, several datasets reporting inertial data are available, enabling, for instance, the development of machine learning applications. A daily and sports activities data set” comprises motion data of 19 daily and sports activities (e.g., sitting, standing, lying on the back and right, ascending and descending stairs, etc.) performed by eight subjects for 5 min [128]. In particular, five sensors were applied to five body areas (i.e., torso, right arm, left arm, right leg, left leg), recording inertial data on nine DoF (i.e., x, y, z accelerometers; x, y, z gyroscopes; x, y, z magnetometers). Similarly, in [129], researchers presented the human activity recognition using smartphones data set; the trials were conducted on 30 participants aged 19 to 48. Each participant used a Samsung Galaxy S II smartphone while engaging in six different activities (walking, walking upstairs, walking downstairs, sitting, standing, and lying). Using the phones’ integrated accelerometer and gyroscope, they recorded three-axial linear acceleration and three-axial angular velocity at a constant rate of 50 Hz. The resulting dataset was divided into two sets at random, with 30% of the volunteers chosen to create test data and 70% of the participants chosen to create training data.

Moreover, datasets including heterogeneous data typologies are available; for instance, MoVi (motion video) is a publicly available dataset, including video and inertial information [130]. In particular, 60 female and 30 male patients were involved, collecting data during 20 everyday and sports movements. The movements were captured throughout five rounds utilizing an optical motion capture system, video cameras, and inertial measurement units (IMU). The dataset includes 6.6 h of IMU data, 9 h of motion capture data, and 17 h of video from four distinct points of view.

With regard to gait monitoring applications, examples of publicly available datasets are the gait in aging and disease database [131], the MIT database [132], and the Georgia Tech dataset [133]. The first one comprises time series of the walking stride interval acquired from fifteen subjects (i.e., five healthy young adults, five healthy old adults, and five older adults with Parkinson’s disease) [131]. On flat ground, the subjects constantly traversed a path free of obstructions. The stride interval was measured using compact force-sensitive resistors inserted into the shoe. An ankle-worn microcomputer was used to sample the analog force signal at 300 Hz using a 12-bit A/D converter while simultaneously recording the data. The interval between foot strikes was then automatically calculated. The datasets discussed above are summarized in Table 6, classifying them from the point of view of the number of participants, the detected parameters, the employed acquisition system, and the suggested application.

## 6. Conclusions and Future Developments

Wearable sensors are a very promising technology for post-rehabilitation and sports-tracking applications. The assessment of a patient’s physical state, the degree of rehabilitation training, and the quality of the rehabilitation effects are crucial in the recovery process where the collection of a series of data (e.g., movements, bending, joint rotation, and so on) is useful to provide important tracking feedback to the user. These data allow for defining the correct therapies and evaluating their effectiveness and patient progress based on suitable protocols that ensure the patients carry out their physiotherapy programs even at home. Moreover, they allow for optimizing the exercises and assessing the athlete’s progress during sports training.

This paper reviewed the state of research on wearable sensors and devices based on different transduction mechanisms focusing on devices at a micro-electromechanical scale. The paper provided an overview of capacitive, piezoresistive, triboelectric, piezoelectric, and inertial transduction methods, distinguishing between wearable sensors for post-operative rehabilitation monitoring and wearable sensors for athletes’ performance monitoring. A comparison of the technologies analyzed was then provided, highlighting the biophysical parameters they can detect and some key features that allow defining the performance of the sensors. We believe that our review paper shows several novelties compared to similar review works reported in the scientific literature. In detail, our review work focuses mainly on biomechanical sensors for monitoring joint movements, lingering on aspects related to their design, fabrication, and characterization [107]. Other review works analyze systems for rehabilitation monitoring by considering the system architecture rather than the sensors used [107]. Additionally, we focused on biomechanical sensors (i.e., piezoresistive, piezoelectric, inertial sensors, etc.) for monitoring joint movements, leaving out other sensor typologies (electrochemical sensors, biosignal detection stages, etc.) [10,107,108,109]. Furthermore, the presented work analyzes sensing systems for detecting both rehabilitation parameters and sports performances; in contrast, other papers deal exclusively with systems for rehabilitation monitoring or sports performance analysis [9,11,107,108]. Furthermore, similar papers discuss wearable sensing systems and other typologies of sensing devices (ambient sensors, portable devices, etc.) or report a general discussion on applying IoT technologies for monitoring sports performance, losing specificity for the considered topic [9,10,107,108,109].

In addition, our review work explores different sensor technologies in-depth (e.g., piezoelectric, piezoresistive, triboelectric) for joint monitoring without dwelling on specific sensor classes (such as inertial sensors) or specific application areas (e.g., knee) [9,11,108,109]. Finally, comparative analyses are presented in our review for each discussed topic, comparing the performance of the discussed devices and providing insights to establish the systems ranked by best performances. This analysis is a fundamental contribution to our review work, not always shown in other similar works [10,11,107,108,109]. Table 7 summarizes the advantages of our review paper compared to similar review works. As evident, the proposed review presents a more complete and in-depth analysis of the leading technologies for monitoring rehabilitation and sports performances, not limiting the discussion to specific sensor categories, applications, or monitored body areas.

With regard to the future perspectives for wearable systems for monitoring rehabilitation and sports performances, several factors must be considered. In addition to the full integration of sensors/actuators, energy sources, procession, and communication within the clothes, future developments of wearable medical systems are facing toward extending the monitoring capabilities, improving the user’s comfort, and making the manufacturing process more simple, scalable, and sustainable. In detail, new textile-based sensor structures could be developed, featuring multiple sensing capabilities, reduced invasiveness, and high reliability. Examples of devices complying with such characteristics are already under development, combining different sensing mechanisms into a textile sensor, providing them with extended sensing capabilities and thus improving their applicability [137,138]. Furthermore, advanced electronic textile solutions must be designed, enabling their integration into fiber-based textile devices, and ensuring improved optical and mechanical capabilities. Such solutions must be characterized by low power consumption or have self-sustainable device features, along with being bio-compatible to avoid irritations caused by their prolongated contact with the skin.

Additionally, new haptic feedback technologies could be developed, including inflatable interfaces and dynamic textile shapes (e.g., moving origami textile structures [139]). These solutions may improve the wearing experience of the wearable device, making it more pleasant during long periods of rehabilitation. In this context, customized design and digital manufacturing may be obtained through 3D scanning and 3D printing technologies, altering their shape and functionality depending on specific demands [140]. The possibility of precisely and comfortably positioning the sensors for specific patients is made possible by customization in design.

Furthermore, textile-based displays for discretely providing visual feedback could be developed and integrated into clothes. Recently, textile-based OLEDs were proposed and their usage in practical situations demonstrated [141]. The textile-based OLEDs demonstrated a consistent lifespan under ambient settings, sufficient mechanical toughness to withstand deformation caused by human movement, and washability, i.e., the ability to keep their optoelectronic capabilities even in wet situations such as rain, sweat, or washing.

## Figures and Tables

**Table 1:** Table showing the main angles on the joints of the upper and lower body limbs.

Symbol	Representation
$\theta_{S-\mathrm{ab}/\mathrm{ad}\;}$	Shoulder abduction/adduction
$\theta_{S-\mathrm{hab}/\mathrm{ahd}\;}$	Shoulder horizontal abduction/adduction
$\theta_{S-\mathrm{in}/\mathrm{ex}\;}$	Shoulder internal/external rotation
$\theta_{W-\mathrm{fl}/\mathrm{ex}\;}$	Wrist flexion/extension
$\theta_{W-\mathrm{ra}/\mathrm{ul}\;}$	Wrist radial deviation/ulnar deviation
$\theta_{F-\mathrm{pr}/\mathrm{su}\;}$	Forearm pronation/supination
$\theta_{E-\mathrm{fl}/\mathrm{ex}}$	Elbow flexion/extension
$\theta_{A-\mathrm{IC},\;\mathrm{front}}$	Ankle initial contact angle—frontal plane
$\theta_{A-\mathrm{IC},\;\mathrm{sag}}$	Ankle initial contact angle—sagittal plane
$\theta_{A-\mathrm{MAX},\;\mathrm{front}}$	Ankle maximum—frontal plane
$\theta_{A-\mathrm{MAX},\;\mathrm{sag}}$	Ankle maximum angle—sagittal plane
$\theta_{K-\mathrm{IC},\;\mathrm{front}}$	Knee initial contact angle—frontal plane
$\theta_{K-\mathrm{IC},\;\mathrm{sag}}$	Knee initial contact angle—sagittal plane
$\theta_{K-\mathrm{MAX},\;\mathrm{front}}$	Knee maximum—frontal plane
$\theta_{K-\mathrm{MAX},\;\mathrm{sag}}$	Knee maximum angle—sagittal plane

**Table 2:** Classification of human physiological parameters usually identified in monitoring patients’ rehabilitation (**a**) and athletes’ activities (**b**).

Rehabilitation Parameters	Sports Activity Parameters
Joint bending (angle or ROM) ^(A)^ [18,21,22,24,25,26,27,28,29,32,39,40,41,42,43,44,50,53,54,55,56,57,58,59,60] ^(^*^)^	Joint bending (angle or ROM) ^(A)^ [18,21,22,24,25,26,27,28,29,32,39,40,41,42,43,44,50,53,54,55,56,57,58,59,60] ^(^*^)^
Joint rotation ^(B)^ [18,21,24] ^(^*^)^	Joint rotation ^(B)^ [18,21,24] ^(^*^)^
Neck muscle vibration (frequency and pattern) [22,27,28,29,30,32,39,42,53]	Jumping (distance and peak acceleration) [39,44,50,55]
Heart parameters (HR—heart rate, HRV—heart rate variability, blood pressure) [21,27,28,30,42,56] ^(^*^)^	Heart parameters (HR—heart rate, HRV—heart rate variability, blood pressure) [21,27,28,30,42,56] ^(^*^)^
Breathing parameters (RR—respiration rate, inhalation/exhalation times, flow rate) [21,29,30,31,32,53,61] ^(^*^)^	Breathing parameters (RR—respiration rate, inhalation/exhalation times, flow rate) [21,29,30,31,32,53,61] ^(^*^)^
Swallowing (interval and frequency) [21,33,34]	Hit acceleration [47]
Chewing (duration and frequency) [21]	Hit pressure [40,43,46,47]
Movement response time ^(C)^ [47] ^(^*^)^	Movement response time ^(C)^ [47] ^(^*^)^
Movement trajectories [47] ^(^*^)^	Movement trajectories [47] ^(^*^)^
Walking (speed and stance time and pressure) [22,29,39,41,43,44,50,55,62] ^(^*^)^	Walking (speed and stance time and pressure) [22,29,39,41,43,44,50,55,62] ^(^*^)^
Plantar pressure distribution [50,62,63] ^(^*^)^	Plantar pressure distribution [50,62,63] ^(^*^)^
	Fatigue [64]
	Running (speed, pace, acceleration) [22,39,41,43,45,50,55,64]
(**a**)	(**b**)

^(^*^)^ It is applied for both rehabilitation and sports activities. ^(A)^ Bending movement around a joint (such as the knee or elbow). ^(B)^ Twisting movement produced by the superposition of small rotational movements around the joint. ^(C)^ Time interval between the stimulus’ appearance and the corresponding muscle reaction’s start.

**Table 3:** Comparison of the main transduction technologies used in wearable systems to monitor sports and rehabilitation activities.

Transduction Technology	Active/Passive	Strengths	Drawbacks	Body Area
Inertial	Passive	Reliability High accuracy Small size Low power Wide operative range Wide bandwidth Linearity	Sensitivity to EMI Sensitivity to undesired body movement Complexity in data processing	Knee [20,54,95], elbow [96,97], chest [97,98], wrist [97,99], shoulder [97], head [97], throat [100]
Piezoresistive	Passive	Robustness Simple structure Low cost Well-known mechanism Stable over time High durability	High power consumption Sensitive to environmental conditions Difficulty in scaling down Poor linearity	Foot [62], wrist [27,30,44,56,60], finger [27,32,44,56,60], throat [27,30,32], chest [31,32], elbow [32,44,60], knee [25,32,44,60], neck [32], nose [30], cheek [27,30], ankle [44], abdomen [60]
Capacitive	Passive	Low cost Wide operative range Wide temperature range	Sensitive to environmental conditions Poor linearity Vibration sensitivity	Finger [28,29,53,55], throat [28,29,53], wrist [28,57], cheek [29], knee [29,55], elbow [29,57], foot [29], belly [53,61], head [46]
Piezoelectric	Active	Active Robustness Simple structure Biocompatibility (according to used material) Simple scaling-down	Responsive only to dynamic stimuli High sensitivity to temperature and moisture	Wrist [21,24,39,87], throat [21,22,25,28,39,87], cheek [21], nose [21,87], shoulder [24], forearm [24], elbow [22,24,39,42], foot [22], knee [39,64], finger [30,33,79], ankle [64]
Triboelectric	Active	Active High sensitivity Adaptable to different sizes and shapes Low cost Simple structure	Difficult signal reading Sensitive to environmental conditions Low durability Limited short circuit output current	Tongue [33], finger [50], wrist [26,50,91], elbow [26,41,50,58], foot [41,50,91], knee [26,41,91], belly [91], ankle [26], shoulder [26], spine [26]

**Table 4:** Specification comparison of analyzed rehabilitation monitoring systems.

Reference	Sensing Technology	Materials	Sensitivity	Range of Detection	Body Area of Application	Measured Parameters	Response Time	Transmission Technology
Ianculescu et al. [54]	IMU	N.A. ^(^*^)^	N.A. ^(^*^)^	N.A. ^(^*^)^	Knee	Knee bending angle	N.A. ^(^*^)^	Wireless
He et al. [29]	Capacitive	MXene (Ti_3_C_2_Tx)/AgNWs	0.01–2.04 kPa^–1 (A)^	0–2 kPa	Throat, cheek, finger, knee, elbow, foot	Voice recognition, air-blowing intensity, joint bending angle, walking frequency, and weight pressure	ms	Wired
Chen et al. [53]	Capacitive	Nickel-coated fabric	N.A. ^(^*^)^	N.A. ^(^*^)^	Belly, throat, finger	Breathing frequency and intensity, voice recognition, finger bending angle	5–50 µs	Wired
Lei et al. [28]	Capacitive	ACC/PAA/alginate hydrogel	0.17 kPa^–1 (A)^	0–1 kPa	Finger, throat, wrist	Finger bending angle, voice recognition, heartbeat	N.A. ^(^*^)^	Wired
Park et al. [61]	Capacitive	PDMS AgNW CFs	0.015 kPa^–1 (A)^	10–50 kPa	Belly	Breathing frequency and intensity	N.A. ^(^*^)^	Wired
Yao e Zhu [55]	Capacitive	AgNW/PDMS Ecoflex	1.62 MPa^–1 (A)^ below 500 kPa	Up to 1.2 MPa	Knee, finger	Finger bending and knee bending frequency	40 ms	Wired
Sheng et al. [57]	Capacitive	GaInSn	N.A. ^(^*^)^	Stretchability up to 250%	Elbow, wrist	Wrist and elbow bend angles	<10 ms	Wireless
Yun et al. [33]	Triboelectric	PET/Cu/PTFE	47 mV/kPa	20–100 kPa	Tongue	Pressure and frequency of tongue movements	N.A. ^(^*^)^	Bluetooth
Tan et al. [62]	Piezoresistive	rGO–Ag	3.90 kPa^–1 (B)^	0–100 kPa	Foot	Pressure distribution on the foot sole	170 ms	Wired
Tognetti et al. [25]	Piezoresistive	Carbon-coated PA and lycra elastic yarns	(Angular sensitivity) 960 Ω/° ^(C)^	N.A. ^(^*^)^	Knee	Knee bending angle	N.A.	Wired
Ge et al. [56]	Piezoresistive	PDMS AgNW	4.29 N^–1 (D)^	0–2 N	Wrist, finger	Heartbeat and finger bending angle	8 ms	Wired
Zhu et al. [32]	Piezoresistive	TPU/CNT–CNC	GF = 321	Stretchability > 500%	Throat, finger, elbow, knee, neck, chest	Voice recognition and finger, elbow, knee, and neck bending degree	N.A. ^(^*^)^	Wired
Dan et al. [30]	Piezoresistive	PDMS/AgNW	14.1 kPa^–1^, 4.8 kPa^–1^, 1.84 kPa^–1 (B)^	(0–3.5) kPa, (3.5–10) kPa (10–40) kPa	Wrist, nose, cheek, throat	Heartbeat, exhalation frequency, facial expression signals, and voice recognition	47 ms	Wired
Kim et al. [27]	Piezoresistive	PUD/CNTs	0.31 kPa^–1^, 0.1 kPa^–1^, 0.03 kPa^–1 (E)^	<1000 Pa, (1–20) kPa, >20 kPa	Wrist, throat, finger, cheek	Arterial and jugular heartbeat, finger bending angle, cheek bulging frequency, voice recognition	36.7 ms	Wired
Lu et al. [44]	Piezoresistive	PANI/PAAMPSA	GF = 1.7 (100% strain) GF = 14.52 (1500% strain)	Stretchability up to 1935%	Wrist, elbow, finger, ankle, knee	Finger, elbow, wrist, and knee bending angle, frequency of walking and jumping	N.A. ^(^*^)^	Wired
Kim et al. [24]	Piezoelectric	PVDF–elastic threads	N.A. ^(^*^)^	0–5 N	Shoulder, forearm, elbow, wrist	Bending and rotation angle of shoulder, wrist, and forearm	N.A. ^(^*^)^	Bluetooth
Wang et al. [21]	Piezoelectric	(P(VDF–TrFE)/MWCNT)	540 mV/N	0.5–5.0 N	Wrist, throat, cheek, nose	Heartbeat, intensity and frequency of breathing, swallowing and chewing	N.A. ^(^*^)^	Wired
Natta et al. [34]	Piezoelectric	AlN/Mo on Kapton	0.025 V/N	10–50 kPa	Throat	Frequency, duration, and latency of swallowing	15 ms	Wireless

(*) N.A. Not Available. ^(A)^ Sensitivity is calculated by normalizing the capacitance variation as ∆CC0P where P is the applied pressure. ^(B)^ Sensitivity is calculated by normalizing the resistance variation as ∆RR0P  where P is the applied pressure. ^(C)^ Angular sensitivity is calculated as ∆R∆α where 𝛼 is the bending angle. ^(D)^ Sensitivity is calculated by normalizing the resistance variation as δ(∆RR0)δ(F) where 𝐹 is the applied force. ^(E)^ Sensitivity is calculated by normalizing the current variation as δ(∆II0)δ(P) where 𝑃 is the applied pressure.

**Table 5:** Specification comparison of analyzed sports activity monitoring systems.

Reference	Sensing Technology	Materials	Sensitivity	Range of Detection	Body Area of Application	Measured Parameters	Response Time	Transmission Technology
Masihi et al. [46]	Capacitive	PDMS	0.3 kPa^–1 ^ 3.2 MPa^–1 (A)^	<50 Pa 0.2–1 MPa	Head	Head pressure distribution	115 ms	Wired
Li et al. [41]	Triboelectric	PDMS–elastic resin	N.A. ^(^*^)^	2–260 N	Elbow, knee, foot	Knee and elbow bending angles, pressure, and frequency of steps in running and walking	N.A. ^(^*^)^	Wired
Yang et al. [50]	Triboelectric	TPU/silicone rubber/conductive fabric	0.054 V/kPa^–1^	2–200 kPa	Finger, wrist, elbow, foot	Finger, wrist, and elbow bending angles; plantar pressure distribution during walking, running tiptoe, and jumping	N.A. ^(^*^)^	Wired
Guo et al. [22]	Piezoelectric	PVDF/BaTiO_3_ (NW)	0.017 kPa^–1 (B)^	1–40 kPa	Foot, elbow, throat	Elbow bending angle, voice recognition, pressure, and frequency of steps in running and walking	290 ms	Bluetooth
Zhao et al. [39]	Piezoelectric	PVDF/Ag/PET	N.A. ^(^*^)^	N.A. ^(^*^)^	Elbow, knee, wrist, finger, throat	Vocal recognition; elbow, wrist, finger, and knee bending angle; walking, jumping, and running frequency	N.A. ^(^*^)^	Bluetooth
Li et al. [64]	Piezoelectric	(PVDF–HFP)/ZnO	1.92 V/kPa^–1^	0.02–0.5 N	Knee, ankle	Frequency and degree of the knee and ankle bending during running	20 ms	Bluetooth
Liu et al. [42]	Piezoelectric	PVDF	N.A. ^(^*^)^	N.A. ^(^*^)^	Throat, elbow, finger	Vocal recognition, finger and elbow bending angles	N.A. ^(^*^)^	Wireless
Saponara et al. [47]	IMU, strain gauge, electro-goniometer	Aluminum	N.A. ^(^*^)^	Hundreds of g (gravity force)	Hip, knee, elbow	Speed, acceleration, pressure, trajectory, and response time of punch and kick	N.A. ^(^*^)^	Bluetooth
Ma et al. [40]	Resistive–capacitive	GO–CNT/PU e-textile	0.1124 kPa^–1^, 0.0283 kPa^–1^, 0.0021 kPa^–1 (A)^	0–9 kPa, 9–37 kPa, 37–110 kPa	Chest, knee	Hit pressure distribution and knee bending angles	120 ms	Wired
Zhu et al. [18]	Triboelectric/ piezoresistive	PTFE/latex–PVDF/hydrogel	N.A. ^(^*^)^	N.A. ^(^*^)^	Wrist	Wrist bending and rotation angles	N.A. ^(^*^)^	Bluetooth
Mariello et al. [43]	Triboelectric/ piezoelectric	PDMS/ Ecoflex–AlN/Mo	59.4 mV/kPa^–1^ 160 mV/kPa^–1^, 3.7 mV/kPa^–1^	0–50 kPa, 50–120 kPa, 120–400 kPa	Foot, elbow, wrist, finger, ankle, knee, neck	Hit pressure on human skin; walking and running speed; finger gestures; ankle, elbow, neck, wrist, and knee bending	N.A. ^(^*^)^	Wired

(*) Not Available. ^(A)^ Sensitivity is calculated by normalizing the capacitance variation as ∆CC0P where P is the applied pressure. ^(B)^ Sensitivity is calculated by normalizing the voltage variation as ∆VVsdP  where P is the applied pressure and V_s_ is the saturation voltage.

**Table 6:** Table summarizing the datasets previously discussed, classified according to the number of participants, the acquisition system, the detected parameters, and the suggested application.

Dataset	Provided by	No of Participants	Parameters	Approach	MoCap System Details	Suggested Application
UI–PRMD [125]	University of Idaho	10	Locations and angular orientations of the body joints	Vision-based	Vicon optical trackers Kinect cameras	Monitoring rehabilitation exercises
KIMORE [126]	Marche Polytechnic University	78	Joint locations	Vision-based	Kinect cameras	Detection motor dysfunction
M. Capecci et al. [127]	Marche Polytechnic University	7	Joint locations	Vision-based	Kinect v1	Evaluation of karate moves
Daily and sports activities data set [128]	Bilkent University	8	Inertial data	Sensor-based	Inertial sensors (25 Hz sampling frequency)	Activity recognition
Human Activity recognition using smartphones data set [129]	University of Genoa	30	Inertial data	Sensor-based	Smartphone (Samsung Galaxy S II)	Activity recognition
MoVi dataset [130]	York University	90	Camera images, joint locations, inertial data	Vision-based Sensor-based	15 cameras (Qualisys Oqus 300 and 310) 2 stationary cameras (RGB Grasshopper2) 2 hand-held cameras (iPhone 7) 17 IMU sensors (Noitom Neuron Edition V2)	Motion recognition
Gait in aging and disease database [131]	PhysioBank	15	Stride interval	Sensor-based	Force-sensitive resistors	Normal gait and Parkinson’s disease analysis
MIT database [132]	MIT	24	View, time	Vision-based	Sony Handycam	Gait recognition
Georgia Tech [133]	Georgia Tech	20	View, time, distance	Vision-based	-	Gait recognition

**Table 7:** Comparison between the presented review work and similar ones reported in the scientific literature.

References	Limitations of Similar Review Papers	Advantages of our Review Paper
L. do Nascimento et al. [134]	- It mainly deals with the architectural aspects of the discussed systems. - It does not consider sensors for monitoring athletic gestures. - It deals with other sensor typologies (electrochemical sensors, biosignal detecting stages, etc.). - It does not present any comparative analyses.	- It deals with the design, fabrication, and characterization of sensors for monitoring rehabilitation and sports performance. - It considers both systems for monitoring rehabilitation and sports performance. - It focuses only on wearable sensing systems. - It presents comparative analyses of the discussed systems.
S. Patel et al. [135]	- It does not consider sensors for monitoring athletic gestures. - It is not focused on wearable systems. - It deals with other sensor typologies (electrochemical sensors, biosignal detecting stages, etc.). - It does not present any comparative analyses.	- It deals with the design, fabrication, and characterization of sensors for monitoring rehabilitation and sports performances. - It focuses only on wearable sensing systems. - It considers only biomechanical sensors. - It presents comparative analyses of the discussed systems.
R. T. Li et al. [136]	- It considers only sensing systems for monitoring sports performances. - It mainly deals with motion-tracking systems based on inertial sensors. - It does not present any comparative analyses.	- It deals with sensing systems for monitoring both rehabilitation and sports performance. - It focuses on the main typologies of biomechanical sensors for monitoring rehabilitation and sports performances. - It presents comparative analyses of the discussed systems.
D. R. Seshadri et al. [10]	- It is not focused on sensing systems for detecting body movements. - It considers only sensing systems for monitoring sports performance. - It does not present any comparative analyses.	- It considers only sensing systems for detecting body movements. - It deals with sensing systems for monitoring both rehabilitation and sports performance. - It presents comparative analyses of the discussed systems.
Y. Zhao and Y. You [9]	- It considers only sensing systems for monitoring sports performance. - It reports a general discussion on sensor technology for motion detection, not dwelling on specific systems reported in the literature. - It analyses IoT-based wearable systems for monitoring sports performance, not focusing on the sensory aspects. - It mainly deals with motion-tracking systems based on inertial sensors.	- It deals with sensing systems for monitoring both rehabilitation and sports performance. - It provides an in-depth discussion of several sensing systems for motion detection reported in the scientific literature. - It deals with the design, fabrication, and characterization of sensors for monitoring rehabilitation and sports performance. - It focuses on the main typologies of biomechanical sensors for monitoring rehabilitation and sports performance.
S. Bahadori et al. [11]	- It focuses on knee-motion detection systems. - It mainly deals with motion-tracking systems based on inertial sensors. - It does not present any comparative analyses.	- It considers sensing systems for monitoring different joints and body movements. - It focuses on the main typologies of biomechanical sensors for monitoring rehabilitation and sports performance. - It presents comparative analyses of the discussed systems.

## Data Availability

Data of our study are available upon request.
